# A Review: Electrode and Packaging Materials for Neurophysiology Recording Implants

**DOI:** 10.3389/fbioe.2020.622923

**Published:** 2021-01-14

**Authors:** Weiyang Yang, Yan Gong, Wen Li

**Affiliations:** Microtechnology Lab, Department of Electrical and Computer Engineering, Michigan State University, East Lansing, MI, United States

**Keywords:** neurophysiology, implantable, microelectrodes, organic, inorganic, packaging, materials

## Abstract

To date, a wide variety of neural tissue implants have been developed for neurophysiology recording from living tissues. An ideal neural implant should minimize the damage to the tissue and perform reliably and accurately for long periods of time. Therefore, the materials utilized to fabricate the neural recording implants become a critical factor. The materials of these devices could be classified into two broad categories: electrode materials as well as packaging and substrate materials. In this review, inorganic (metals and semiconductors), organic (conducting polymers), and carbon-based (graphene and carbon nanostructures) electrode materials are reviewed individually in terms of various neural recording devices that are reported in recent years. Properties of these materials, including electrical properties, mechanical properties, stability, biodegradability/bioresorbability, biocompatibility, and optical properties, and their critical importance to neural recording quality and device capabilities, are discussed. For the packaging and substrate materials, different material properties are desired for the chronic implantation of devices in the complex environment of the body, such as biocompatibility and moisture and gas hermeticity. This review summarizes common solid and soft packaging materials used in a variety of neural interface electrode designs, as well as their packaging performances. Besides, several biopolymers typically applied over the electrode package to reinforce the mechanical rigidity of devices during insertion, or to reduce the immune response and inflammation at the device-tissue interfaces are highlighted. Finally, a benchmark analysis of the discussed materials and an outlook of the future research trends are concluded.

## Introduction

Neurological disorders and diseases in the central and peripheral nervous systems, such as Parkinson's disease, Alzheimer's disease, and epilepsy, are affecting hundreds of millions of people worldwide (Siuly and Zhang, 2016; Feigin et al., 2019; Wijeratne et al., 2020). Neurophysiology recording electrodes act as a seamless interface between the nervous system and the outside world and help diagnose these neurological diseases. Several types of neural signals could be measured from the brain using electrodes (Hashemi Noshahr et al., 2020), including electroencephalogram (EEG) (10–400 μVpp; 1 mHz−200 Hz) (Acharya et al., 2019), electrocorticogram (ECoG) (10–1,000 μVpp; 1 mHz−200 Hz) (Thukral et al., 2018; Kanth and Ray, 2020), in addition to local field potentials (LFPs) (0.5–5 mVpp; 1 mHz−200 Hz) and action potential spikes (50–500 μVpp for extracellular; 10–70 mVpp for intracellular; 100 Hz−10 kHz) (Herreras, 2016; Chen et al., 2017a). EEG is noninvasive but suffers from low spatial resolution and poor signal-to-noise ratio (SNR) because of signal attenuation through the scalp and skull. Mechanical disturbances and electromyographic activities also incur the artifacts that further influence the spatial and temporal resolutions of EEG recording (Jiang et al., 2019). Unlike EEG, ECoG directly measures the signals from the cerebral cortex via neurophysiological implants without any internal and external source noises due to the scalp and skull, leading to lower tissue interference, greater precision, higher sensitivity, and reduced noise interference. Although some special ECoG arrays, such as “NeuroGrid,” have been proved to be capable of recording spike activity and LFPs (Khodagholy et al., 2015), almost ECoG can only gather the electrophysiological signals from the superficial surface of the cerebral cortex and is incapable of capturing spikes from individual neurons. Therefore, penetrating electrodes suitable for recording LFPs and action potentials with high spatiotemporal resolution have been widely used in the neuroscience community (Hong and Lieber, 2019). Despite recent advances in electrode technologies, all existing electrode implants are still suffering from poor long-term stability and crosstalk, due to long-standing challenges such as material biocompatibility, hermetic packaging, the relatively large physical dimensions of the devices, as well as mechanical mismatch between the brain tissue and the implant (Fattahi et al., 2014). Similarly to the central nervous system, for the peripheral nervous systems, surgically implanted neural electrodes could be categorized into regenerative electrodes, intra-fascicular electrodes, inter-fascicular electrodes, and extra-neural electrodes (Russell et al., 2019). These electrodes have more strict requirements for some material properties, such as flexibility and biocompatibility (Russell et al., 2019). Indeed, careful selection and design of electrode and packaging/substrate materials are significantly essential to improve the recording quality and long-term stability of the electrode implants. Therefore, to thoroughly study the electrical activity of neuronal circuits underlying various disorders, developing innovative neural recording devices have been long-standing interests of many scientists, intending to achieve the best combination of excellent electrical properties, high spatiotemporal precision, prominent biocompatibility, outstanding long-term stability, and safety of the electrode devices.

To date, many research efforts have been devoted to the design and fabrication of implantable neural recording electrodes with different materials on various substrates. The materials of these devices could be classified into two broad categories: electrode materials as well as packaging and substrate materials. While silicon-based materials, as well as common metallic materials (e.g., platinum or iridium) and their derivatives (e.g., platinum black and iridium oxide), are widely used in electrode manufacturing, they are still antagonistic to the soft, ionic, wet, and dynamic nature of the biological tissue, with their hard, electronic, dry, and static nature. Non-conventional conducting materials that were not initially developed for neural implants have been receiving much attention and applied for neurophysiological recording in recent years because of their favorable properties and manufacturing advantages. Examples of these emerging electrode materials include graphene (Park et al., 2016; Kostarelos et al., 2017), indium tin oxide (ITO) (Aydin and Sezgintürk, 2017), carbon-polymer hybrid nanostructures (Guo et al., 2017; Saunier et al., 2020). In the search for suitable packaging and substrate materials, various types of glass and ceramic materials, such as alumina (Shen and Maharbiz, 2019), silicon nitride (Zhao et al., 2019), silicon carbide (SiC) (Lei et al., 2016), and silica (Cheng et al., 2013b), have greatly expanded the options for researchers. With the advancement of material synthesis technology, polymers have played an important role in medical device packaging. With their stable and unique physical properties, many polymeric materials, including SU-8 (Altuna et al., 2010), polyimide (Bakonyi et al., 2013), Parylene (Ceyssens and Puers, 2015), polydimethylsiloxane (PDMS), and liquid crystal polymers (LCPs) (Hwang et al., 2013), have been widely used as packaging materials for neural recording electrodes. The design consideration of neural stimulation electrodes is similar to that of neural recording electrodes, concerning biocompatibility, mechanical properties, electrical properties, and stability (Shepherd et al., 2018). For example, platinum black and Ir/IrOx are also widely used as stimulating electrodes (Zhang et al., 2015; Chen et al., 2019). Large charge storage capacity is specifically required for simulating electrodes to achieve better stimulating performance (Hudak et al., 2017). Neural stimulators also have the same strict requirements on hermeticity, long-term stability, and biocompatibility of device package (Vanhoestenberghe and Donaldson, 2013; Donaldson and Brindley, 2016). Many materials that have been utilized in neural stimulating probes include but are not limited to: ceramics, glass, epoxy, silicone, and so on (Amanat et al., 2010; Vanhoestenberghe and Donaldson, 2013; Shepherd et al., 2018).

To draw a clear picture and guide the material design for future device development, this article reviews the current materials for the fabrication and packaging of neural recording implants that were reported in the literature in the most recent years. In the following sections, Section Key Challenges of Neural Implants discusses several important material properties, including electrical properties, mechanical properties, stability, biodegradability/bioresorbability, biocompatibility, and optical properties, as well as the critical impact of these properties on the performance of electrode implants. Section Key Material Characteristics provides a detailed discussion of various electrode materials in three different categories: inorganic materials (e.g., metals and semiconductors), organic materials [e.g., poly(3,4-ethylene dioxythiophene):poly(styrene sulfonate (PEDOT:PSS) and poly(pyrrole) (PPy)], and carbon-based materials (e.g., graphene and carbon nanostructures). Approaches to improve the recording performance of the electrode materials are also reviewed. Next, Section Electrode Materials categorizes and introduces various solid and soft packaging materials, respectively. Also highlighted are the biopolymers for coating and surface functionalization to temporarily enhance the mechanical rigidity of the implants during insertion or to suppress the immune response and inflammation at device-tissue interfaces. Finally, the conclusion and outlook in Section Packaging and Substrate Materials provides an insightful overview of the discussed electrode and packaging materials and put forward the future and potential research trends in the related fields.

## Key Challenges of Neural Implants

### Tissue Responses

**Figure 1:**
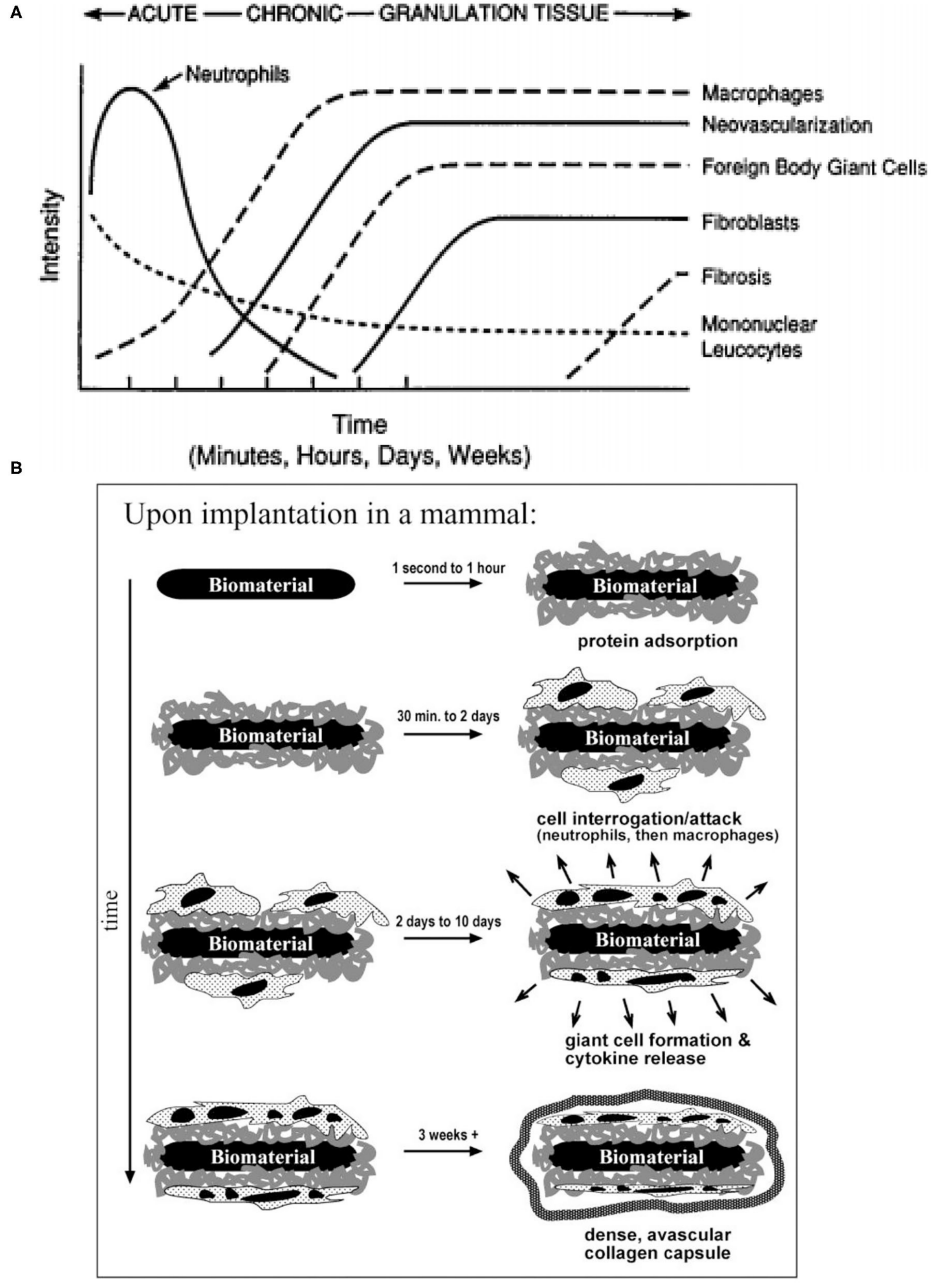
Temporal variations and stages of tissue responses to neural implants. **(A)** The temporal variations in the acute inflammatory response, chronic inflammatory response, granulation tissue development, and foreign body reaction to implanted biomaterials (reprinted with permission from Anderson, 2001). **(B)** The different stages of foreign body reaction to an implanted neural implant (reprinted with permission from Ratner and Bryant, 2004).

Before selecting candidate materials for neural electrode implants, it is essential to understand the biological response to foreign objects, e.g., neural implants. The inflammatory response is usually caused by tissue injured during the implantation surgery or the existence of the implants in the body. Inflammation achieves the purpose of containing, neutralizing, diluting, or isolating the harmful substances through a series of complex physiological reactions (Anderson, 2001). These inflammatory reactions will significantly affect the functionality and stability of implanted devices. First, acute inflammation will occur in the first few days of implantation. A large amount of blood will flow to the damaged tissue through the dilated blood vessels, and then a blood clot will be formed to close the wound (Anderson, 2001). Then the tissue fluid containing water, salt, and protein will form edema (Anderson, 1988). At this stage, the implants have to overcome the contamination of blood and tissue fluids that may cover the implants and cause device malfunction. Similarly, the extrusion and tissue deformation that may be caused by edema also require a certain strength of the inserted implant. This means that the electrode, package, or substrate materials must have a certain mechanical strength. The tissue environment is moist and chemically rich, which is not an ideal environment for implants (Shen and Maharbiz, 2020). Moreover, the immune response will release reactive oxidative species (ROS), which attack and degrade the implants (Patrick et al., 2011; Takmakov et al., 2015). With the continual presence of the implant, the inflammatory response will be transformed into chronic inflammation. A major feature in this phase is the regeneration of damaged epithelium and vascular tissue (Wahl et al., 1989; Fong et al., 1990; Pierce et al., 1991), which may encapsulate the implants and consequently degrade the recording stability and accuracy of the electrodes. The immune response of the tissue does not stop at this phase, so the implant still faces the attack of ROS. Once a foreign object is implanted into the body, a sequence of events (e.g., inflammation and foreign body response) occurs in the surrounding tissue and ultimately ends at the formation of foreign body giant cells at biotic-abiotic interfaces (Anderson et al., 2008). The intensity of the response is directly related to the properties of the implant (Anderson, 2001), such as size, shape, topography, and chemical and physical properties of the selected material. As the final stage of the inflammatory response, tissues try to wrap the implants with a vascular, collagenous fibrous capsule with a thickness of 50–200 μm to isolate foreign objects (Ratner and Bryant, 2004). This fibrous wall will undoubtedly affect the electrical coupling between the implant and the targeted neurons, which may cause signal degradation and ultimately implant failure. The temporal variations of tissue responses and stages of foreign body reaction are shown in Figures 1A,B.

### Surgical Challenges

Before the neurophysiology recording implants are surgically implanted into the body (Morales and Clément, 2018), sterilization is a significant and indispensable step to reduce the microbial contaminants (e.g., viruses) by six orders of magnitude (Stieglitz, 2010), and thereby reduce the intensity of inflammation. Various sterilization methods have been explored to suitably match various neurophysiology recording implants (Stieglitz, 2010). Nowadays, there are a great number of sterilization methods compliant with biomedical device regulation (Booth, 1998), including chemical sterilization (ethanol 70%), dry heating (160–190°C), autoclaving (120–135°C), ethylene oxide gas, hydrogen peroxide gas plasma, peracetic acid and UV radiation. Relatively required high temperatures in dry heat and autoclaving sterilization will accelerate the oxidation and corrosion speed of the electrode materials, and hence can destroy the functionality of the whole implants, especially for easily-oxidized materials, such as silver thin films and silver nanowires (Elechiguerra et al., 2005; Chu et al., 2019). For packaging materials, high temperature and liquid uptake are the main concerns during these sterilization procedures (Lecomte et al., 2018; Shen and Maharbiz, 2020). In particular for biodegradable packaging materials, dry heat and autoclaving sterilization may cause partial denaturation to collagen (Wiegand et al., 2009), morphology change to silk (Yucel et al., 2014), and melting and degradation to [poly(lactic-co-glycolic acid) PLGA] (Athanasiou et al., 1996). The sterilization process has less impact on synthetic polymer packaging materials than biodegradable materials, but it is still worthy of note. For instance, significant delamination of Parylene C encapsulation has been revealed after the steam sterilization process because of the insufficient adhesion strength between Parylene C and encapsulated device (Schander et al., 2016). In addition, because its glass transition temperature is around 90°C, high-temperature may cause degradation in the mechanical and optical properties of Parylene C. Ceramic materials have relatively broad options of sterilization methods due to their low water-vapor permeability and high-temperature resistance (Shen and Maharbiz, 2020). While ethylene oxide sterilization can be operated at relatively low temperatures, the permeability of polymers can allow liquid stored in the buck material and a degassing step is required (Shen and Maharbiz, 2020). In addition, ethylene oxide is a central nervous inhibitor, stimulant and protoplasmic toxin (Mendes et al., 2007). Improper exposure of neural implants to ethylene oxide can cause acute poisoning and chronic effects, such as severe headache, loss of consciousness, neurasthenic syndrome and dysfunction of the vegetative nerve with long-term light exposure (Golberg, 2018). Unlike ethylene oxide gas, hydrogen peroxide gas plasma has the benefit of non-toxic final decomposition products (McEvoy and Rowan, 2019). However, because of the oxidation reaction during the sterilization of hydrogen peroxide gas plasma (McEvoy and Rowan, 2019), selecting electrical materials should be more careful to avoid damages due to excessive oxidation.

## Key Material Characteristics

### Electrical Properties

For electrophysiology recording, the electrode/electrolyte boundary is comprised of electrochemical reactions (Faradic) and double-layer charging (capacitive) (Eles et al., 2018; Ferro and Melosh, 2018). Electrochemical impedance (typically at 1 kHz) is a critical factor in benchmarking the performance of the recording electrodes (Szostak et al., 2017). The targeted impedance range of microelectrodes is from ~0.1 to 2 M*Ω* with the proper recording system utilization (Neto et al., 2018). Although some studies indicate the impedance does not have a major impact on the signal quality (Arcot Desai et al., 2010), most studies state that electrochemical impedance greatly affects the signal recording quality (Chung et al., 2015; Kozai et al., 2015; Zhao et al., 2016). The design of electrodes present tradeoffs in dimensions, electrochemical impedance, and background noise of recording. Miniaturized electrodes with diameters of 4 to 100 μm allow for single-unit recording with high spatial resolution and minimal invasiveness, but at the expense of increased electrochemical impedance that could cause signal quality reduction and background Johnson noise increase. In particular, Johnson noise, also known as thermal noise, is proportional to the square root of the impedance of electrodes (Fang et al., 2015; Wang et al., 2018), as given by the following general equation:

$$\begin{array}{l} V_{noise}=\sqrt{4kTRe\left\{Z\right\}\Delta F} \end{array}$$

**Figure 2:**
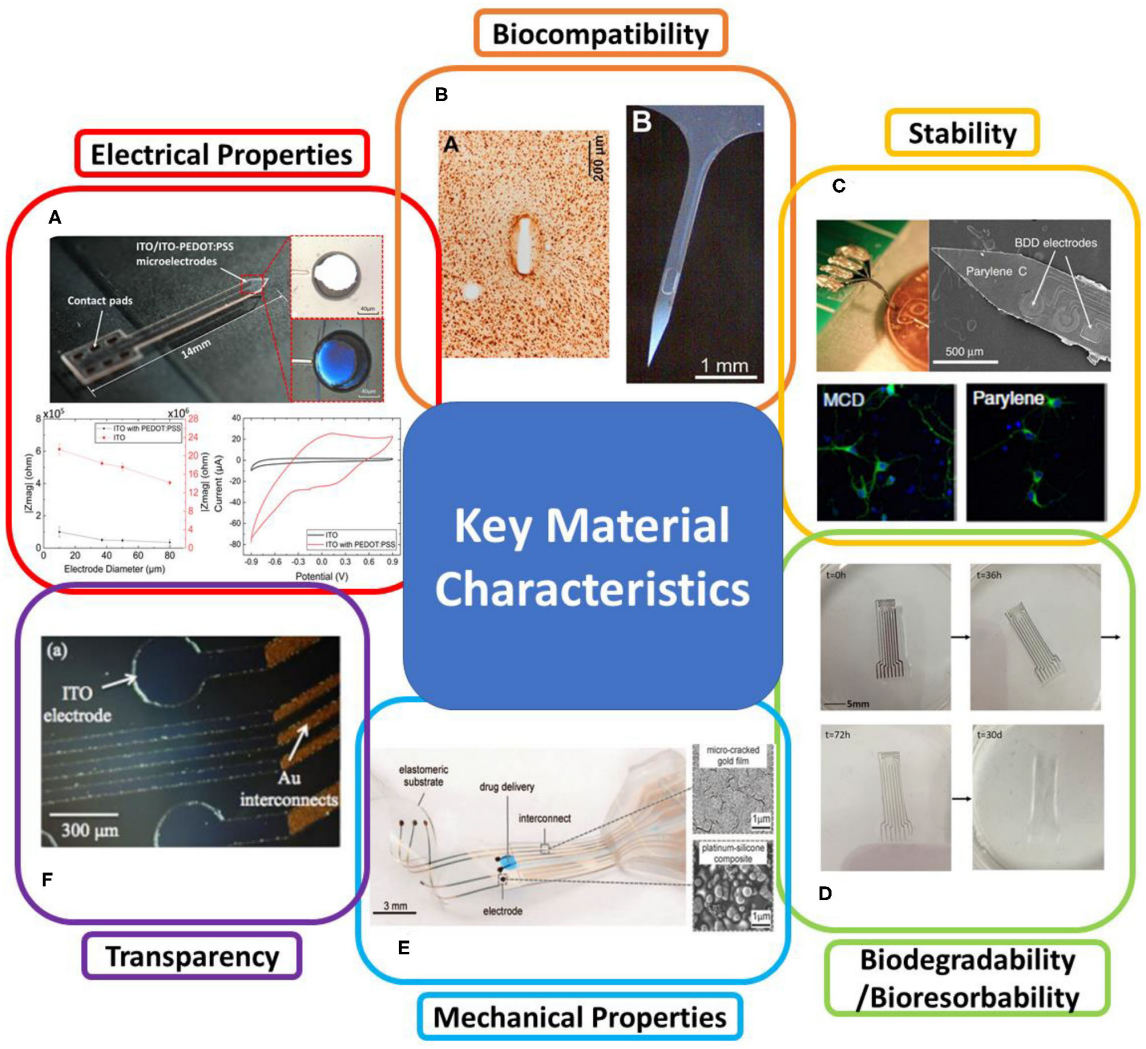
Key material characteristics of neural recording implants. **(A)** Electrical properties. The example shows a neural recording probe (upper) with ITO (white) /ITO-PEDOT:PSS (blue) microelectrodes. The added PEDOT:PSS has been proved to improve electrochemical impedance (lower left) and charge storage capacity (lower right) due to the increased surface roughness (reprinted with permission from Yang et al., 2017). **(B)** Biocompatibility. The example shows the neuronal preservation and the severity of astrogliosis (left) around implanted SU-8 devices (right) (reprinted with permission from Márton et al., 2020). **(C)** Stability. The example shows an electrode array made of mechanically and chemically stable, boron-doped polycrystalline diamond (BDD) (upper). Morphological response of rat cortical neurons on the Parylene C and microcrystalline diamond (MCD) substrates (lower) appeared similarly to the control substrate (reprinted with permission from Fan et al., 2020). **(D)** Biodegradability and bioresorbability. The example indicates patterned molybdenum (Mo) electrodes on the resorbable substrate (PLLA/PCL) (reprinted with permission from Xu et al., 2019). **(E)** Mechanical properties. The example shows a mechanically flexible neural implant consisting of soft platinum-silicone composited electrodes (upper right) and micro-cracked gold film (lower right) interconnect on a silicone substrate (reprinted with permission from Minev et al., 2015). **(F)** Optical transparency. The example shows a transparent ITO ECoG implant combined with optical stimulation (reprinted with permission from Kwon et al., 2013).

Where k is Boltzmann's constant, T is the temperature value, Re{Z} is the resistive component of the electrode impedance, and ΔF is the frequency band (Stenger and McKenna, 1994). The most common solution to this challenge is to increase the effective surface area of microelectrodes by surface modification with electrically conducting polymers, nanomaterials, or nanostructures (Baranauskas et al., 2011; Xie et al., 2012), which will effectively reduce the impedance while keeping device dimensions at a cellular scale to achieve high recording resolution, as shown in Figure 2A. Conducting polymers (CPs), such as PPy and poly (3,4-ethylenedioxythiophene) (PEDOT), has also shown promise in improving ionic-to-electronic charges transfer at the interface between the tissue and the recording site (Bobacka et al., 2000; Cui et al., 2001), therefore increased charge capacity of microelectrodes. Insulation layer as a part of the recording system, once it has been damaged due to material degradation or insulation delamination (Beygi et al., 2019), the electrical properties of the entire system will also change. The delamination changes electrode electrical properties by expanding the geometric area of the exposed conductor, in turn, this averages the recorded potentials across an electrode surface area and attenuates the neural signal (Wellman et al., 2018). Besides, an increase in the surface area will cause abnormal impedance change of the electrode during long-term implantation (Gong et al., 2020), which will further deteriorate the recording quality (Prasad et al., 2012).

### Biocompatibility

The biocompatibility of a recording electrode implant depends on various factors, including electrode materials, device geometry, and surrounding environments. From the material standpoint, biocompatibility can be defined as the “ability of a material to perform an appropriate host response in a specific application” (Williams, 1986). An ideal biomaterial for neural recording implants should be non-cytotoxic *in vivo* and release no substances or substances at only low, non-toxic concentrations. The tissue should produce minimal glial encapsulation surrounding the implant and only mild foreign body reaction without evidence of necrosis or implant rejection (Navarro et al., 2005; Márton et al., 2020), as shown in Figure 2B. Evaluation of material/device biocompatibility is critical and may include the tests of cytotoxicity, acute/chronic systemic toxicity, sub-acute/sub-chronic toxicity, sensitization, irritation, genotoxicity, hemocompatibility, toxicokinetic studies, and immunotoxicology (Feron et al., 2018). Since the same material may respond differently to different biological environments, the International Organization of Standard (ISO) enacts various test and evaluation protocols to evaluate the materials' biocompatibility, considering various body contact types, contact time, environments of intended use (*in vitro, ex vivo*, or *in vivo*), and test methods as mentioned in Hanson et al. (1996) and Frederick (2007).

### Stability

Material stability is another important consideration of neural recording implants (Tang et al., 2008; Lago and Cester, 2017; Li et al., 2018a; Chiang et al., 2020). The fabrication imperfection of the electrode or the packaging materials, such as unavoidable pinholes and defects, could cause the oxidation and delamination of the materials, and hence, shorten the longevity of the implants in liquid environments with a high concentration of ions, such as cerebrospinal fluid (Porrazzo et al., 2014; Chen et al., 2017b). The heterogeneous junction where an electrode interfaces with an adhesion-promoting layer (e.g., Ti or Cr) or the heterogeneous alloys is also a potential risk of electrode reliability. The two different metals can form a short circuit galvanic cell in the tissue fluid that accelerates the corrosion of one of the metals and weakens the metal-to-metal bonding strength (McFadden, 1969). Therefore, higher atomic weight transition metals with high corrosion resistance, such as platinum and iridium, were selected as the primary electrode materials (Cogan et al., 2005; Rodger et al., 2008; Patrick et al., 2011). Homogenous alloys with multiple metal elements can also improve corrosion resistance (Wellman et al., 2018). Surface modification of electrodes with electrodeposited CPs is another method to slow down metal corrosion and improve device stability (Pranti et al., 2017; Dijk et al., 2020). For example, electrodeposited PEDOT is quite chemically stable in the damp, oxygen-rich environments because PEDOT can be further polymerized by the oxygen and protect the metal electrodes from direct exposure to reactive, oxygenated solution (Halliwell, 1992), and therefore, prevent the metals from corrosion (De Vittorio et al., 2014; Yang et al., 2019a). However, further polymerization could cause the increased electrochemical impedance of the whole electrodes due to cracking or delamination of the PEDOT layer (Kozai et al., 2014; Wellman et al., 2018).

Biofouling also contributes to the instability of the neurophysiological recording implants. Biofouling leads to the encapsulation of protein and glial cells on electrodes, especially on those with high electrochemical surface areas, and therefore, restricts ionic diffusion at the electrode-electrolyte interface (Seymour and Kipke, 2007; Du et al., 2015). In addition, the tissue response persistently promotes the degradation of electrode materials and insulation. To minimize electrode biofouling, significant efforts have been made on surface modification or functionalization to alter the chemical terminations, morphology, and wettability of the electrode surface (Wellman et al., 2018). Several hydrogel and polymer coatings, such as polyethylene glycol (PEG) and PEG methacrylate (PEGMA), have been utilized to improve the hydrophilicity of the electrode surface (Justin and Guiseppi-Elie, 2010; Heo et al., 2012; Cheng et al., 2013a). With large amounts of water in their structures, these materials are highly hydrated to increase the energetic penalty of removing water for protein and microorganism attachment. Engineered antifouling electrode materials, such as *sp*^3^ carbon-enriched, boron-doped polycrystalline diamond (BDD), also show the advantages of improved biocompatibility and reduced biofouling compared to conventional electrode materials (Meijs et al., 2016; Fan et al., 2020), as shown in Figure 2C. Moreover, nanostructured surfaces with low friction and low surface energies can effectively decrease cell attachment onto the implant surface, and hence, reduce the possibility of biofouling formation (Chapman et al., 2017; Boehler et al., 2020).

### Biodegradability/Bioresorbability

In contrast to stability, biodegradability is another prevailing topic that has been extensively studied in neural implants (Thukral et al., 2018). Unlike the aim of the stability to keep the implant devices *in vivo* for long-term detection, biodegradability requires the implants to be biodegradable and bioresorbable after a certain period (days to weeks) in order to avoid secondary damage to surrounding tissues during implant removal (Won et al., 2018). Some inorganic materials, including metals [e.g., gold nanoparticles (GNPs)], semiconductors [e.g., silicon nanomembranes (Si NMs)], and dielectrics [e.g. silicon dioxide (SiO_2_)], have shown outstanding degradation behavior (Kang et al., 2016; Lu et al., 2018). Combining those materials with biodegradable organic materials enables high-performance and less-invasive implantable devices (Li et al., 2018b). Despite studies on biodegradable bulk materials, recently, special attention has been paid to engineering multi-functional thin-film materials that combine degradability with other desired properties (electrical, optical, mechanical) and can be dissolved in the phosphate-buffered saline (PBS) in 30 days (Wu et al., 2014; Xue et al., 2018; Xu et al., 2019), as shown in Figure 2D. However, the biodegradation performance of most thin-film degradable materials has only been tested in de-ionized (DI) water or saline solution (0.9% NaCl) (Lewitus et al., 2010, 2011). Since the *in vivo* environments are much more complicated than the *in vitro* environments due to the presence of biological molecules, such as proteins and cells, *in vivo* evaluation of these materials must be conducted to understand better their degradation rate and safety in living tissues (Lecomte et al., 2017; Lee et al., 2017b).

### Mechanical Properties

Mechanical properties of the neural implants are extremely important for *in vivo* applications. The Young's moduli of traditional solid materials (silicon, glass, and metal) range from 50 to 200 GPa, orders of magnitude higher than those of the nervous tissues that are typically 3.15–10 kPa (Patil and Thakor, 2016). The mechanical property mismatch between the soft tissue and the stiff implants induces reoccurring electrode movement from the target neurons in response to natural body motions (Gilletti and Muthuswamy, 2006), resulting in unreliable recording from the same neurons for an extended period. In the long term, the presence of stiff implants elicits the effect of tissue staining at the implant site due to inflammatory response, and consequently neuronal degeneration and glial scar formation near the electrodes that prohibit the transformation of neural signals (Fang et al., 2015; Lacour et al., 2016; Ferro and Melosh, 2018; Wang et al., 2018). Moreover, the stress induced by the micromotions of surrounding tissues can cause mechanical damage to the implants, such as cracks or delamination of the electrode materials, and then permanent device failure (Cogan et al., 2004; Marin and Fernández, 2010; Patil and Thakor, 2016). Compared to solid materials, soft materials, such as silicone, Parylene C (PA), SU-8, and polyimide (PI), with Young's moduli of 1–10 GPa, are more compliant with the soft tissue to form a conformal contact (Wang et al., 2012; Minev et al., 2015; Patil and Thakor, 2016), as shown in Figure 2E. PDMS can achieve even lower Young's modulus of 1 MPa, becoming one of the softest prevailing packaging and substrate materials for neural implants (Sun et al., 2004).

Besides the above materials with consistent mechanical properties, shape-memory materials can be deformed from the initial shape under external stimuli, such as temperature, humidity, etc. (Lee et al., 2016a). Before and during implantation, devices made of shape-memory materials are stiff enough to penetrate the target tissue (Beattie et al., 2000; Christensen et al., 2014). Once adapted to the physiological conditions, the implanted devices can be programmed to snake around and climb nerves (Moore, 2019). For example, Zhao et al. reported a 16-electrode microwire electrode arrays made of a shape memory metallic alloy (Zhao et al., 2018), nitinol, which an equiatomic alloy of nickel and titanium exhibiting shape memory effect due to thermally-induced phase transition (Lendlein and Kelch, 2002). The device can conform to the brain vasculature with minimized damage to the blood vessels during implantation. Shape-memory polymers (SMPs), such as thiol-ene/acrylate-based SMPs (Ecker et al., 2017; Black et al., 2018a), provide good elasticity and the diminished rigidity and mechanical mismatch with the soft tissue, suitable for use in manufacturing surgical devices and medical implants. The shape-memory effect of these materials is induced by the cross-links of polymeric chains and the corresponding external stress at the transition temperature (Lee et al., 2016a).

### Optical Transparency

Optical transparency of an electrode implant allows one to combine electrophysiological recording with other modalities, such as high-resolution optical imaging and optogenetics (Won et al., 2018). To date, high-resolution, systematic electrophysiological recording on optically scanned tissue surfaces of the brain has not been implemented, because conventional opaque electrode materials do not satisfy the optical qualification of high-resolution imaging (Fekete and Pongrácz, 2017). Optogenetics applications also require high transmittance of the materials over a broad spectrum or under the specifically targeted wavelength for activating or inhibiting the genetically modified neurons with the minimum optical propagation loss (Thukral et al., 2018). With a unique combination of electrical conductivity, broadband transparency, and biocompatibility, several transparent conducting materials, such as ITO (Figure 2F), graphene, and PEDOT:PSS, have been explored as electrode materials (Park et al., 2018). These materials also provide sufficiently wide bandgaps to limit photoelectrochemical (PEC) artifacts that arise from photo illumination of electrodes during opto-stimulation and two-photon imaging (Castagnola et al., 2017; Kostarelos et al., 2017; Yang et al., 2017). In addition, Au nanomesh electrodes (Seo et al., 2017) or PEDOT:PSS-coated Au (Qiang et al., 2018) microelectrodes have been proven to achieve low electrochemical impedance and some degree of optical transparency, capable of electrophysiological recording in the brain. To realize fully transparent neural recording implants, polymers, such as polyethylene terephthalate (PET), PA, and PDMS, usually act as transparent substrate and encapsulation of the electrodes (Kim et al., 2017; Ren et al., 2018).

## Electrode Materials

### Inorganic Materials

Recently, much attention has been devoted to investigating innovative electrode materials to improve electrical, mechanical, and optical properties, as well as stability, biocompatibility, or biodegradability of recording electrodes (Fattahi et al., 2014). This section classifies the electrode materials into inorganic, organic, and carbon-based materials, and discusses the advantages, disadvantages, and applications of each specific material in detail.

#### Metals

Metals are the most prevailing and common electrode materials for neural recording for nearly 50 years (Kim et al., 2018). Widely used metal electrode materials, such as Au, platinum (Pt), iridium (Ir), tungsten (W), and tantalum (Ta), offer a great number of desirable properties, including chemical inertness, high electrical conductivity, and excellent biocompatibility in biological environments (Barrese et al., 2016; Won et al., 2018; Burton et al., 2020). Au/Pt and Ir/Pt have been used as the electrode materials for “Utah array” and “Michigan Probe,” two of the most popular neural interface electrodes (House et al., 2006; Kim et al., 2010a). However, these materials suffer from limited electrochemical conductivity and injection charge density, especially when the electrode is shrunk to a micrometer scale for better spatial resolution (Lee et al., 2016a).

To address the impedance-size trade-off in microelectrodes, three dimensional (3D) nanostructured Au microelectrodes have been developed wherein nanoporous structures were created on the microelectrode surface to achieve larger surface area and therefore lower impedance (Fairfield, 2018). The nanotopography of such nanoporous structures also improves *in vivo* stability of electrode implants by reducing the incidence of glial scar encapsulation while maintaining high neuronal coverage. Surface modification with Au nanorods, nanoflakes, or nanopillars is another option to increase the effective recording area without change the overall electrode dimensions (Zhou et al., 2009; Kim et al., 2010b; Nick et al., 2014). For example, Nick et al. fabricated Au nanopillars on the microelectrodes, showing a reduction of 1 kHz impedance by up to 89.5 times and dramatic impedance decrease over 1 Hz to 100 kHz (Nick et al., 2014). With a determined diameter, a larger high-aspect ratio of the nanopillars results in lower impedance of the electrode. Similarly, Zhou et al. integrated an Au-nanorod array on flexible thin-film microelectrodes using locally patterned anodized porous alumina as a template (Zhou et al., 2009). The interface impedance of this 3D electrode was 25 times smaller than that of conventional two dimensional (2D) planar microelectrodes under the same dimensions. Moreover, 3D electrodes modified with Au nanoflakes have also been reported by Kim et al., demonstrating a maximum impedance reduction factor of 57.9 with an electrode diameter of 5 μm (Kim et al., 2010b).

**Figure 3:**
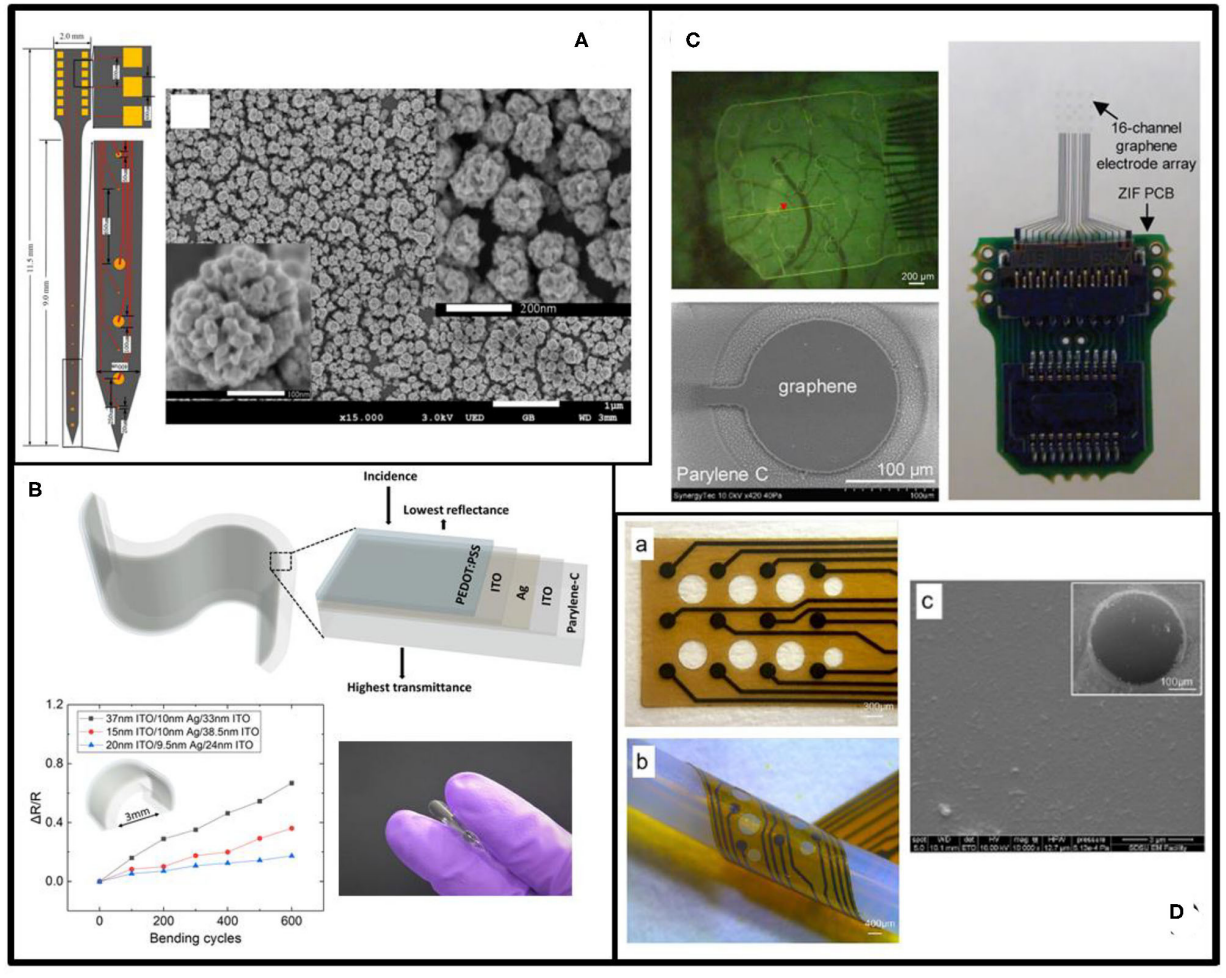
Examples of electrode materials. **(A)** The electrodes of the neurophysiological implants (left) are made of Au/Pt alloy as indicated in the SEM image (right) (reprinted with permission from Zhao et al., 2016). **(B)** Flexible PEDOT:PSS-ITO-Ag-ITO thin films on Parylene C substrate (upper) for ECoG array to overcome the brittleness of a single layer of ITO. The lower left and lower right figures show the good bendability and flexibility of ITO-Ag-ITO structure, respectively (reprinted with permission from Yang et al., 2019b). **(C)** Transparent graphene as the electrode material (lower left) on the μECoG array (right) for the neurophysiology signal recording and imaging (upper left) (reprinted with permission from Park et al., 2018). **(D)** Thin-film glassy carbon recording electrodes on flexible polyimide (left). The SEM image (right) shows the surface morphology of the glassy carbon (reprinted with permission from Vomero et al., 2017).

An alternative nanostructure for electrode surface modification is Pt black, a nanoparticulate-like Pt formed from electroplating. Zhang et al. show that, with Pt black, the 1 kHz impedance of a 100 μm diameter electrode wire decreased from 16.6 to 3.5 k*Ω* and the charge injection limit increased from 0.286 to 1.906 mC/cm^2^ (Zhang et al., 2015). Furthermore, alloys with two or more than two metals have been investigated for nanoparticle synthesis to improve the stability of metallic nanoparticles. One example is the bimetallic Au/Pt alloy nanoparticle modified Au microelectrode, which exhibits an average 1 kHz impedance of 0.23 M*Ω* with a recording site of 20 μm diameter (Zhao et al., 2016), as shown in Figure 3A.

Ir/IrOx (iridium oxide) is another prevailing electrode material and often used in the format of either a bulky wire or a thin film coating (Zeng et al., 2017; Black et al., 2018b; Chen et al., 2019; Ghazavi et al., 2020). Ir wires are very stiff and highly resistant to corrosion (Loeb et al., 1995), whereas IrOx thin films are unstable and prone to degradation as electrode dimensions decrease and charge densities increase (Cogan et al., 2004). However, untreated Ir electrodes suffer from limited charge injection capacity. Ir alloys, such as PtIr, exhibit significantly improved mechanical and electrochemical properties (Wellman et al., 2018). Cassar et al. electrodeposited a PtIr coating (EPIC) on the tip of 75 μm-diameter microwire electrodes, resulting in reduced electrochemical impedance from 534 ± 57 k*Ω* to 80 ± 18 k*Ω* and improved SNR (Cassar et al., 2019).

#### Semiconductors

Semiconductors can be readily configured into various electronic elements (e.g., sensors, transistors, switches, etc.) with desired properties (e.g., signal transduction, amplification, multiplexing, etc.) to achieve a complex, integrated biointerface system (Maiolo et al., 2019; Zhang et al., 2020). Organic semiconductors provide unique advantages of mechanical compliance, biodegradability, and stretchability. Contrastly inorganic semiconductors are more rigid but provide faster response, higher sensitivity, better accuracy, and lower power consumption of biological sensing than organic semiconductors because of high charge carrier mobilities in inorganic materials (Jiang and Tian, 2018). Moreover, advancement in modern semiconductor technologies allows precise batch fabrication of high-performance inorganic semiconductor devices in various architectures at submicron or even nanometer scale, matching the size of subcellular and molecular targets.

Silicon (Si) is the most commonly used semiconducting material in neurophysiological implants. The well-developed microfabrication and photolithography techniques for complementary metal-oxide-semiconductor (CMOS) integrated circuits enable the design and fabrication of high density, high-channel-count multielectrode arrays, capable of mapping activity from large-scale neural networks with high spatiotemporal resolution (Hong and Lieber, 2019). As the current state of the art, the Neuropixel Si probe developed by Jun et al. integrates 960 recording sites (384 configurable recording channels) on a 70 × 20 μm shank, weighs only ~0.3 g, and provides on-chip signal amplification and digitization (Jun et al., 2017). Each probe enables stable and chronic recordings from more than 100 neurons for over 150 days while remaining low noise (Jun et al., 2017).

With high sensitivity to changes in electrical potentials and surface charges, Si-based nanostructure materials are also used to make low impedance microelectrode interface for neurophysiology recording (Fairfield, 2018; Jiang and Tian, 2018). For example, Si nanowires have been utilized as low impedance nanoelectrodes to intracellularly record actional potential from cultured neurons at high precision (Robinson et al., 2012; Liu et al., 2017a). Besides, a forest of randomly oriented gold coated-Si nanowires has been proved to achieve the non-invasive extracellular recording of astrocytes by mimicking the properties of astrocytes *in vivo* (Saracino et al., 2020). Compared to bulky materials, improved stretchability and bendability can be achieved with Si nanowires. Similarly, an amorphous atomic structured Si material has been proposed to create mesostructures with fibrils and voids, with an average Young's modulus of 2–3 orders smaller than that of the single-crystalline Si (Jun et al., 2017). As key building blocks, nanowires can also be integrated with microporous gel-based scaffolds, yielding highly sensitive and flexible 3D neural probes for mapping the propagation of the action potential (Dai et al., 2016). These 3D electrodes offer excellent spatial resolution and stability with little immune response to chronic implantation. In addition, Si nanowires can be configured into field-effect transistors (FETs), capable of sensing neurophysiological signals at a faster switching speed. Unlike faradaic measurement of neural signals through electrodes, the charge carrier density of FETs can be modulated as a function of LFP in surrounding tissues, allowing spikes tracking along neurites and neural networks with single-cell resolution and reasonably high sensitivity (Hutzler et al., 2006; Patolsky et al., 2006; Veliev et al., 2017). Recently, Yu et al. reported a flexible and bioresorbable neural electrode array based on Si NMs (Yu et al., 2016). With biodegradable SiO_2_ insulation and PLGA substrate, the whole device was able to degrade in PBS (pH = 10) within 15 days.

ITO is a well-known n-type semiconductor material that is often utilized in transparent microelectrodes. ITO has high conductivity, excellent transparency over the entire visible spectrum due to a large bandgap of around 4 eV, as well as confirmed biocompatibility (Falco et al., 2016). ITO can be grown on either solid or flexible substrates using well-developed physical vapor deposition techniques (e.g., sputtering). However, similar to metals, ITO electrodes suffer from increased electrochemical impedance when the electrode sizes decrease, leading to undesirable electrochemical reactions with the brain tissue and poor recording quality due to increased thermal noise and ion-based electric fluctuations of surrounding media (Yang et al., 2017). In addition, ITO is relatively brittle, making it unsuitable for use in large patterns (e.g., pads or interconnection wires) on flexible substrates (Kwon et al., 2013). Surface modification of ITO with conductive thin film coatings (e.g., PEDOT:PSS, Ag, Au) has been explored to address these shortcomings. Recently, Yang et al. reported an ultra-flexible, conductive, and transparent thin film using a PEDOT:PSS/ITO/Ag/ITO multilayer structure on PA, as shown in Figure 3B. The electrode showed at least 10× reduction in electrochemical impedance, ~7% transmittance improvement, and stability after over 600 cycles of mechanical bending (Yang et al., 2019b). Other semiconducting materials, such as germanium (Ge), silicon germanium alloy (SiGe), indium-doped zinc oxide (IZO), indium-gallium-zinc oxide (a-IGZO), and zinc oxide (ZnO), has also been investigated as recording electrode materials because of their desired electrical, mechanical, optical, biocompatible, and stable/biodegradable properties (Gao et al., 2012; Dagdeviren et al., 2013; Lee et al., 2015; Gutierrez-Heredia et al., 2017; Mao et al., 2018; Huerta et al., 2019).

### Organic Materials

Given the same device dimensions, organic materials offer lower Young's moduli than inorganic materials, reducing potential adverse outcomes including inflammation response, glial scar encapsulation, unstable neural recording, and mechanical failure of implants (Lago and Cester, 2017). Organic materials also provide significant advantages of easily modifiable surface structures, mixed ionic and electronic charge transport, less biofouling/surface oxides, and the wide option of biocompatible materials (Feron et al., 2018).

#### Conducting Polymer (CP)

CPs, as organic polymers, consist of monomeric compounds linked in chains of alternating single and double bonds, and doped with a stabilizing counter-ion. CPs have the mechanical properties matched with those of biological tissues. Because conjugated polymers have narrower band gaps, electrons can move easily between the conducting band and valence band. CPs can transduce ionic currents to electronic currents through redox reaction in bulk and volumetric charging, resulting in low impedance and high charge storage capacity (Green and Abidian, 2015; Rivnay et al., 2016). Due to the diversity and adaptability of synthetic processes, the ionic-electronic transport and biochemical surface characteristics are tunable for improving the performance and stability/biodegradation of CPs (Rivnay et al., 2017). Furthermore, dopants, such as small cations/anions (Na^+^, Cl^−^, and ${\text{ClO}}_{4}^{-}$) and large polymeric species (polystyrene sulfonate and polyvinyl sulfonate), can be utilized to improve the electrical conductivity of organic materials by adding electrons to the conduction band (n-doping) or removing electrons from the valence band (p-doping) (Le et al., 2017).

PEDOT:PSS is a prevailing class of CPs for neural interfacing applications. PEDOT:PSS possesses many desirable properties, including high biostability, outstanding biocompatibility, and excellent electrochemical properties. Studies show that, with the same electrode area, the electrochemical impedance of microelectrodes is an order of magnitude lower than that of Pt microelectrodes (Ganji et al., 2017). Khodagholy et al. proposed a PEDOT:PSS-based, high-density NeuroGrid that consists of patterned PEDOT:PSS electrodes with the neuron-size density, capable of simultaneously recording LFPs and action potentials in anesthetized and awake human subjects (Khodagholy et al., 2015, 2016). The enhancement in electrochemical conductivity of PEDOT:PSS-coated electrodes can be attributed to the increased surface roughness of the electrode, as confirmed by Yang et al. (2017, 2019a). Their studies show that the average surface roughness (Ra) of the PEDOT:PSS coated electrode increased from 0.85 nm to 3.33 nm, resulting in dramatically improved charge storage capacity and impedance by several orders of magnitude. Similarly, Pranti et al. reported that electropolymerization of 1 μm thick PEDOT:PSS on chronic Au microelectrodes increased the electrode surface area, and the corresponding electrochemical impedance was reduced by ~99% (Pranti et al., 2018). Besides planar films, ordered PEDOT nanostructures can be self-assembled on the electrode surface with surfactant molecules as a template to further reduce the electrode impedance (Yang et al., 2005). Abidian et al. also reported that PEDOT-based nanotubes enable ~8 times lower impedance and much higher charge capacity density than planar PEDOT films, mostly due to the increased surface area (Abidian et al., 2010). PEDOT:PSS can be applied by spin-coating or ink-jet printing in a low-cost and rapid fashion, but at the expense of poor adhesion with underlying electrode materials. Electrodeposition techniques, such as electroplating, can improve the bonding strength at the PEDOT-electrode interface, preventing potential risk of PEDOT delamination in the biological environment (Abidian et al., 2010). A recent study by Boehlet et al. also demonstrates that pre-treating the smooth Pt electrode with porous Pt structures before the PEDOT deposition can enhance the adhesion between PEDOT and Pt. The PEDOT film deposited on the porous Pt substrate shows no delamination after more than 100 days in accelerated aging tests in PBS (Boehler et al., 2017).

Besides PEDOT, several other CPs, such as PPy, poly(aniline) (PANi), poly(thiophene) (PT), and some of their derivatives (Juarez-Hernandez et al., 2016; Kojabad et al., 2019; Nagane et al., 2020) are also alternative candidates. PPy has outstanding water solubility (Kojabad et al., 2019), 40–200 S/cm conductivity (Guimard et al., 2007), low Yong's moduli of 0.35 psi for thin films (15–35 μm thick) (Diaz and Hall, 1983), and 430–800 MPa for nanocomposites (Sevil and Zuhal, 2010). PPy can be electrodeposited *in situ* on the electrode surface with different dopants. PANi has an electrical conductivity of 5 S/cm (Guimard et al., 2007) and is primarily used as a coating material on electrodes instead of a standalone electrode material due to its relatively small Young's modulus (2–4 GPa) (Passeri et al., 2011). Nanostructured PANi can be synthesized by chemical oxidative or electrochemical polymerization in an aqueous solution that contains a variety of surfactants to precisely tailor the structure of the film at small length scales for increased effective surface area (Yang et al., 2005; Juarez-Hernandez et al., 2016). Functionalized PT copolymer, with precisely tunable electrical, optical, mechanical, and adhesive properties, is also applicable for neural recording electrodes (Nagane et al., 2020). For PT, the maximum conductivity is 10–100 S/cm, and Young's modulus of thin films is ~3 GPa (Wang and Feng, 2002).

### Carbon-Based Materials

Carbon-based materials, such as graphene, carbon nanofibers, carbon nanotubes, are another promising class of electrode materials. Carbon-based materials have high biocompatibility and valuable mechanical properties, such as high tensile strength, and can be prepared by various approaches, including chemical vapor deposition (CVD), electrospinning, and exfoliation.

#### Graphene

Graphene, a 2D single-layer sheet of carbon atoms in a hexagonal arrangement, has a great number of outstanding properties: ~90% optical transmittance (Park et al., 2014), 76*Ω*/ sheet resistance (for a 4-layer structure), 200,000 cm^2^/VS electron mobility (Bolotin et al., 2008), and ~5 × 10^3^ W/mK thermal conductivity (Balandin et al., 2008; Wang et al., 2017; Armano and Agnello, 2019). The remarkable biocompatibility makes graphene an appropriate choice for neural interface applications (Park et al., 2016; Liu et al., 2017b; Thunemann et al., 2018). Moreover, the outstanding transparency of the graphene microelectrode enables simultaneous neurophysiological recording, light stimulation, and optical imaging of living tissues (Park et al., 2014). Despite many benefits, graphene has a large Young's modulus (~1.0 TPa) (Shin et al., 2012; Patil and Thakor, 2016) and a large impedance at the graphene-electrolyte interface, possibly due to the intrinsic hydrophobicity of graphene (Chen et al., 2013). The comparatively low double-layer capacitance of single- or few-layered graphene could cause considerable thermal noise and low SNR of neural recording. Therefore, it is critical to reduce the mechanical mismatch between graphene electrodes and surrounding tissues as well as to improve the electrical properties of hydrophobic graphene. Small area graphene can be prepared using mechanical exfoliation, which is tedious and time-consuming. CVD allows growing high-quality graphene over large areas at either high temperatures of over 1,000°C or on specific substrates in a specific gas mixture, but is incompatible with polymer materials (Kireev et al., 2016). Significant efforts have been made in recent years to transfer CVD graphene from rigid substrates onto soft substrates. For example, Park et al. transferred and stacked four graphene monolayers sequentially onto a flexible PA film (Park et al., 2014) as the electrode material. Later, the same group reported a transparent carbon-layered 16-channel array and succeeded in simultaneous *in vivo* recording of light-evoked neural signals in conjunction with fluorescence imaging (Park et al., 2018), as shown in Figure 3C. The photoelectrochemical effect (also known as Becquerel effect) of graphene is neglectable due to its metal-like zero band nature and relatively high work function (4.5 eV) (Park et al., 2016, 2018). Similarly, Chen et al. transferred graphene onto SU-8 and demonstrated that introducing hydroxyl groups on the graphene surface by a mild stream plasma treatment can effectively increase the water contact angle from 91.1° ±5.6° to 41° ±4.7° (Chen et al., 2013). The increase in graphene hydrophilicity leads to impedance reduction from 7,216 to 5,424 *Ω*/mm^2^ and SNR improvement from 20.3±3.3 dB to 27.8±4.0 dB. Besides electrode configurations, Kireev et al. developed graphene-based FETs on flexible polyimide-on-steel and found that the device did not show significant loss in recording capability after up to 1,000 cycles of mechanical bending (Kireev et al., 2016).

#### Carbon Nanostructures

3D carbon nanostructures, such as carbon fibers (CFs) and carbon nanotubes (CNTs), can be utilized as a standalone electrode or as a surface coating to improve the surface area and electrochemical impedance (Kozai et al., 2012; Fattahi et al., 2014; Patel et al., 2016, 2017; Fairfield, 2018). Standalone carbon fiber microelectrodes (CFMEs) are typically constructed by insulating carbon nanofibers with pulled glass pipettes (Hejazi et al., 2020) or PA (Guitchounts et al., 2013; Patel et al., 2015; Deku et al., 2018; Gillis et al., 2018; Massey et al., 2019) followed by opening the electrode tip with chemical etching, plasma removal, or laser cutting. Recently Patel et al. assembled 16 CFMEs to form a multichannel CFME array, capable of chronic recording of single unite activity for one month (Patel et al., 2015). Such CFMEs electrodes can be functionalized with electrodeposited PEDOT (Patel et al., 2015; Massey et al., 2019) or IrOx (Deku et al., 2018; Gillis et al., 2018) to further improve their impedance and charge capacity density. An alternative method to fabricate CF electrodes is thermal drawing (Guo et al., 2017), by which carbon nanofiber (CNF) composites were unidirectionally aligned in cyclic olefin copolymer (COC) as a recording electrode. The as-fabricated fiber had overall dimensions of <100 × 100 μm^2^, including a single recording site of CNF composite with a size ranging from 18 × 11.3 μm^2^ to 35.2 × 20.1 μm^2^, and dramatically reduced impedance magnitude by 2 orders compared to the conventional polymer electrodes (Guo et al., 2017). Alternatively, Yu et al. demonstrated *in situ* growth of vertically aligned carbon nanofibers on pre-patterned Ni catalyst using direct current catalytic plasma-enhanced CVD. The array consists of 40 electrodes in one line with 15 μm spacing along a complete length of 600 μm. The conical shape of the CNFs facilitates the penetration of electrodes into the interior of tissues or individual cells to improve electrical coupling (Yu et al., 2012). Besides purer CNFs, Saunier et al. reported a composite PEDOT:CNF material combining PEDOT with CNFs through electrochemical deposition. The PEDOT:CNF modified microelectrode demonstrates low specific impedance of 1.28 M*Ω* μm^2^ at 1 kHz and unrivaled charge injection limit of 10.03 mC/cm^2^, suitable for multifunctional electrophysiological recording and neurotransmitter sensing. Moreover, CNF has magnetic susceptibility close to water and tissues, making it compatible with high field functional magnetic resonance imaging (fMRI) to enable high-resolution electrophysiological measurements and anatomical studies of large-scale neural networks without electrode interference with MRI images (Lu et al., 2019).

Unlike the CNFs, CNTs have smaller sizes with higher density and can be divided into single-walled carbon nanotubes (SWCNTs) and multi-walled carbon nanotubes (MWCNTs) (Fattahi et al., 2014). SWCNT is a single graphite sheet wrapped into a cylindrical tube, while the MWCNTs nest several SWCNTs together concentrically, looking like rings of a tree trunk (Zhang et al., 2011). Perfect SWCNTs have outstanding mechanical properties and electrical properties, quite similar to the perfect MWNTs due to the weak coupling of nanotubes in MWNTs (Eatemadi et al., 2014). Additionally, the rolling direction of the SWCNTs decides the properties that are more like metals or semiconductors (Saifuddin et al., 2013). For use in neural electrode implants, CNTs can be electrochemically coated on the conventional tungsten and stainless steel wires under ambient environments at low temperatures to improve the impedance and charge transfer properties of the electrodes (Keefer et al., 2008). Furthermore, the tungsten wires can be etched electrochemically to obtain pure carbon nanotube probes as intracellular recording electrodes (Yoon et al., 2013). Besides electrochemical deposition, CVD methods can synthesize CNTs directly on the tip of quartz-insulated platinum/tungsten electrodes (Ansaldo et al., 2011). Compared to electrochemically deposited CNTs, the chemical vapor deposited CNTs show remarkable mechanical toughness and stability over time. The CVD-CNT-coated microelectrodes can retain unaltered impedance values after 1 year storage or after being subjected to a million current pulses at charge injection limit. CNT can also be integrated with flexible polymer substrates to implement flexible CNT electrodes. For example, Lin et al. embedded pre-patterned CNT structures into a PA film to create a flexible CNT electrode array with significantly reduced mechanical rigidity and low impedance for the high-quality recording of spontaneous spikes from the crayfish nerve cord (Lin et al., 2009). Similar to graphene, studies show that the electrical properties of the CNT-based electrodes can be improved by tuning the hydrophilicity of CNTs. For example, plasma/UVO_3_ treatment of <10s can alter the surface wettability of CNT from superhydrophobicity to superhydrophilicity, mainly due to the formation of -OH terminations (Chen et al., 2010; Su et al., 2010; Pan et al., 2016). Amino-functionalization of the MWCNTs surface with a 2 wt% 1,4-diaminobutane solution can also improve the hydrophilicity of the surface, lasting for at least 6 months in the air (Yen et al., 2011). While widely used in neural electrode implants, the cytotoxicity of these nanostructures is still a big concern, since the nanomaterials can penetrate through the blood-brain barrier (BBB) and cause irreversible cell death and damage to the brain (Tang et al., 2008; Furtado et al., 2018).

#### Glassy Carbon

Glassy carbon (GC) offers a wide range of mechanical, electrical, and electrochemical properties, which can be specifically tailored with different pyrolysis temperatures under different fabrication conditions to match the properties of the target tissue (Cassar et al., 2019). Because flexible polymer substrates are unable to tolerate high pyrolysis temperatures, pattern transfer techniques are often used to fabricate GC based, flexible ECoG microelectrode arrays on polyimide substrates (Vomero et al., 2016; Castagnola et al., 2018), as shown in Figure 3D. Furthermore, coating GC based microelectrodes with CPs, such as PEDOT:PSS, helps to reduce the impedance magnitude of a 60 μm-diameter electrode by at least 2 orders (Vomero et al., 2016). Most recently, Chen et al. designed and fabricated a cone-shaped glassy carbon neural electrode array using 3D printing and chemical pyrolysis technologies (Chen et al., 2020). The electrode had a 0.78 mm^2^ recording area exposed at the tip, and the corresponding impedance, capacitance, and SNR are 7.1 k*Ω*, 9.18 ${\text{mF}/\text{cm}}_{,}^{2}$ and 50.73 ± 6.11, respectively (Chen et al., 2020).

#### Diamond

In recent years, diamond has emerged as a promising electrode material for neurophysiological recording and neurotransmitter sensing. Boron-doped polycrystalline diamond (BDD) offers unique properties, including wide aqueous potential window, chemical inertness, capability for surface modification, tunable electrical conductivity, and biocompatibility (Alcaide et al., 2016; Hébert et al., 2016; McDonald et al., 2017; Yang and Narayan, 2019). Despite the many benefits of this material, the mechanical property mismatch between BDD (Young's module of ~10^3^ GPa) (Wild and Wörner, 2004) and soft tissues is a major obstacle that impedes the development of BDD into fully implantable electrochemical devices. Compared to other semiconducting materials, diamond processing and patterning are more difficult due to its extreme mechanical hardness, lack of ductility, and weldability (Garrett et al., 2016). Therefore, attempts have been made to develop new material synthesis and processing methods to fabricate diamond-based electrodes with improved flexibility. For example, Fan et al. demonstrated a wafer-scale fabrication method to transfer large-scale, pre-patterned BDD microelectrode arrays from a solid silicon substrate onto a flexible PA substrate (Fan et al., 2017, 2020). The electrodes made of the BDD growth side exhibited a rougher topology, a higher *sp*^3^ content, and a large grain size than the nucleation side, enabling a wide working potential window, a low background noise, a resistance to chemical fouling, and a reduced electrochemical impedance (Fan et al., 2020).

## Packaging and Substrate Materials

### Comprehensive Consideration of Packaging/Substrate Materials

For all implantable devices, the biocompatibility of packaging and substrate materials is a prerequisite that must be met (Madou, 2018), not only for the device's long-term stability but also for the user's safety (Onuki et al., 2008). The Implant-induced inflammatory response is complicated and inevitable since the chemical aggressive reaction produced by the inflammatory response is the body's natural self-protection mechanism. Although the impact of the inflammatory response on the performance and lifetime of the implant package needs further characterization by researchers, the aggressive environment caused by inflammation sets a high bar for hermetic seal and chemical stability of the packaging material.

**Figure 4:**
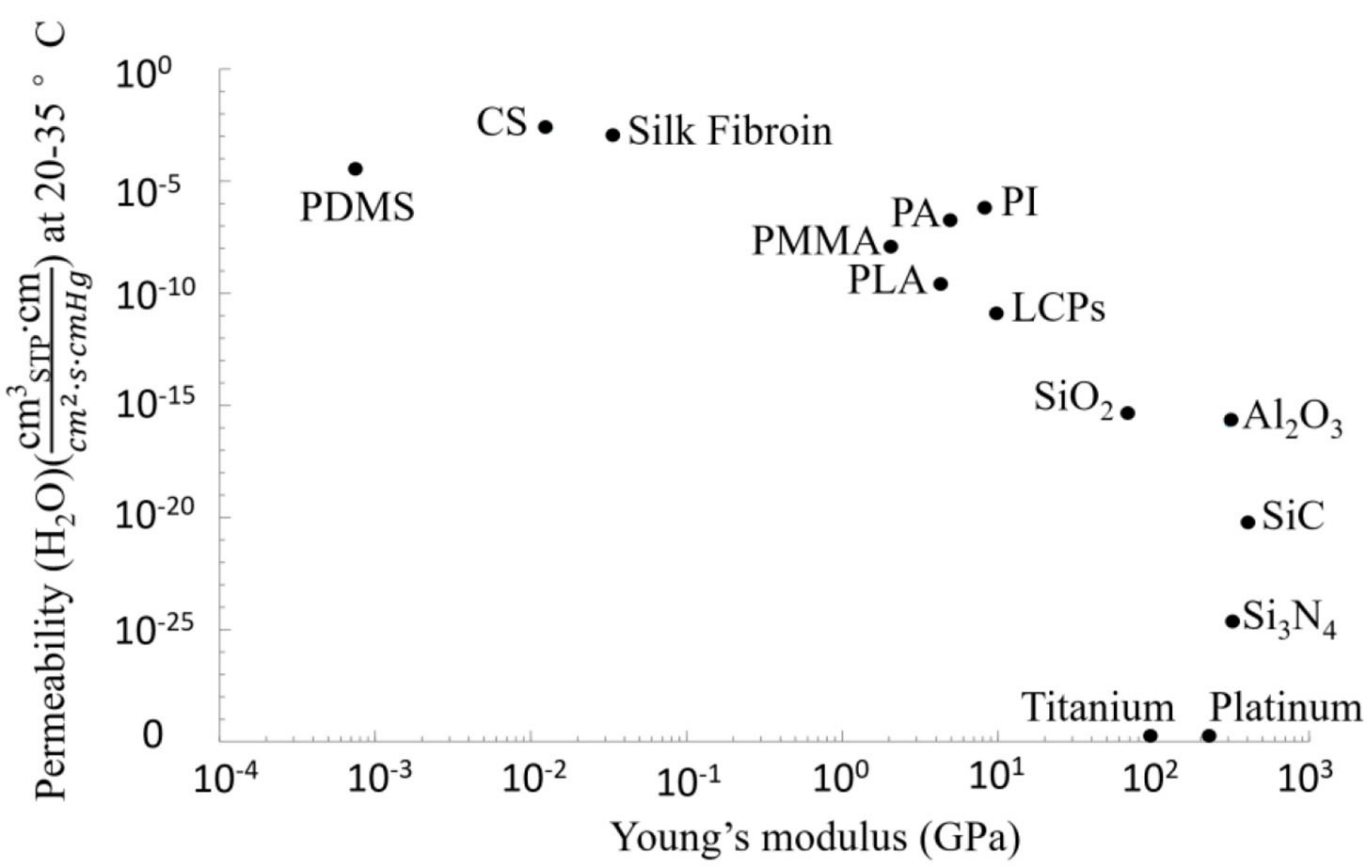
Logarithmic plot of Young's moduli and moisture permeability (H_2_O) for various packaging materials. Data points are representative values available from Table 2.

The next factor to be considered is the hermeticity of packaging materials. There are two basic packaging strategies: hermeticity and non-hermeticity packaging. The choice of a specific packaging strategy depends on the required implant's stability (long-term or short-term) and the inner design (Alt et al., 2016). Due to the complex and aggressive environment *in vivo*, hermeticity is a key criterion for packaging materials of implantable electrodes. Ideally, the packaging should effectively isolate the internal electronics from the human body environment (Joung, 2013), trap the outgassing of the inner materials, and dissipate the electrically-induced heat to the surroundings. The hermeticity of the packaging material directly affects the life expectancy of the implants (Jiang and Zhou, 2009), and can be characterized using permeability. In general, helium permeability is quantified by the amount of helium through a certain thickness of common materials in a certain period (Greenhouse, 1999; Joung, 2013). The helium leak test was recognized as an industry standard (Costello et al., 2012) and can be simply converted into the leak rate of another gas of interest, such as H_2_O (Jeong et al., 2016). However, the helium leak test can be misleading in the case of a polymer package (Vanhoestenberghe and Donaldson, 2011). Therefore, many researchers started to choose moisture permeability as the standard for quantifying the hermeticity of the packaging (Sim et al., 2017; Bettinger et al., 2020; Patil et al., 2020; Song et al., 2020). In theory, all materials will leak to some extent (Ely, 2000) but with different permeabilities. As shown in Figure 4, the permeability of metal is the lowest, which means even a thin (10^−4^ cm) metal can prevent moisture permeation (with a permeability of <10^−25^ $\frac{cm_{STP}^{3}•cm}{cm^{2}•s•cmHg}$) for a very long time (10 years), while the sealing performance of soft polymers, such as silicone, is not good among common packaging materials (Song et al., 2020). As such, thin-film polymers may not be a favorable candidate for impermeable barriers in chronically implanted devices (Jiang and Zhou, 2009). Thicker polymer protective encapsulation or composite materials combining polymers with other materials of better permeability (e.g., metal, ceramics, glass, etc.) should be considered (Jiang and Zhou, 2009).

Other considerations for packaging and substrate materials are based on intended applications and implantation sites. For example, packaging materials for ECoG recording electrodes have a high demand for flexibility and stretchability but low constraint in hermeticity. Therefore, polymers (e.g., silicone and polyetheretherketone), even with relatively high water vapor permeability, are still widely used as packaging and substrate materials in many ECoG implants (Henle et al., 2011b; Mestais et al., 2014; Woods et al., 2018). For recording from deep brain regions, good mechanical strength is required to allow device insertion to the target location with minimal disturbance to the surroundings (Connolly et al., 2015). Therefore, extra attention should be made to the buckling force and the dimensions of the electrode implants, such as silicon probes, to ensure sufficient mechanical strength and toughness for device implantation (Hetke et al., 2002). Packaging of such rigid implants usually involves solid materials, such as SiO_2_ (Cheng et al., 2013b; Lee et al., 2013b) and Si_3_N_4_ (Oh et al., 2003; Zhao et al., 2019).

There are many more factors that limit the choice of materials, including but not limited to, low coefficient of friction of the material to avoid wear debris (Patel and Gohil, 2012), compatibility with wireless communication (Joung, 2013), thermal conductivity, and matched thermal expansion coefficient (Jiang and Zhou, 2009). In general, a major challenge in designing and fabricating a chronically stable neural interface is producing a conformal, dense barrier layer for encapsulation (Joshi-Imre et al., 2019) without releasing toxicity to the tissues (Shen and Maharbiz, 2020). This is particularly difficult when a neural implant has complicated topography (e.g., 3D structure) (Joshi-Imre et al., 2019). While looking for suitable materials, it is also critical to identify various causes of packaging failures under the complex biological environment (Anderson, 2001), which can be attributed to a combination of factors including packaging delamination, inflammatory response, and package damage related to the defects from manufacturing (Joshi-Imre et al., 2019; Gong et al., 2020). No material is perfect, wisely choosing material based on different devices and making good use of the advantages of different materials is a challenge that every engineer must face when designing biomedical implants. “The design of biocompatible materials for device packaging is arguably as much of a challenge as the design of the device itself.” (Wasikiewicz et al., 2013).

### Solid Packaging Materials

In this article, solid packaging materials generally refer to materials whose Young's moduli are higher than cortical bone (15–30 GPa). Most of these materials are inorganic materials represented by metals, ceramics, and glass. Compared to polymers, inorganic solid materials have low gas and moisture permeability, and therefore, have been widely used as substrates and packaging materials in many implantable systems (Loeb et al., 2000; Strojnik and Peckham, 2000; Forde and Ridgely, 2006). Moreover, because of their rigid physical properties, these materials can provide extra mechanical support for device insertion into tissue. However, most inorganic packaging materials cannot fulfill flexibility and mechanical robustness simultaneously. Among all the solid packaging materials, ceramics and glass are very mature packaging materials because of their excellent chemical stability and good hermeticity. Although metal and silicon are not mainstream packaging materials, they still have many favorable characteristics. This section will review and discuss the use of these materials in neural recording devices.

As one of the oldest materials, metal has been utilized in implantable devices for a very long time. Some metal materials, such as titanium, platinum, some alloys, and stainless steel, have good biocompatibility, *in vivo* stability, and very low permeability (Scholten and Meng, 2015). Although few neural recording implants directly use metal as a packaging layer, many applications combined metal thin film with ceramic or polymers to form a hybrid package for implants, such as in a miniaturized inductively-powered neural implant (Khalifa et al., 2017). The advantage of metal lies in its excellent mechanical strength, making it less fragile than ceramic and glass (Scholten and Meng, 2015). However, the application of metal materials to packaging has several problems. First, most metals have good conductivity, which may cause short circuits in internal electronic components. Second, metal corrosion in ionic biological environments is still a challenge (Subramanian et al., 2011). Passive electrochemical corrosion, crevice corrosion, and active electrochemical corrosion can severely affect the stability of metals. Changes in local pH values due to water electrolysis or active electrochemical reactions are also a major threat to metal (Jiang and Zhou, 2009). Third, the bonding strength at the metal-glass interface can be weakened after temperature cycling (Jiang and Zhou, 2009). Moreover, the opacity of metal packages to electromagnetic (EM) field provides EM shielding for internal electronics but restricts their use in wireless implantable devices.

Compared to polymers, many ceramics are gas/water-impermeable, chemically stable, biocompatible, electrically insulating, and physically hard (Vlasov and Karabanova, 1993; Piconi and Maccauro, 1999). However, it is difficult to machine ceramic and glass materials using conventional microfabrication techniques. As such, how to ensure the hermetic seal while allowing the electrode to pass through the ceramic encapsulation must be taken into design consideration (Stieglitz, 2010). In some early devices (Cameron et al., 1997; Loeb et al., 2000), glass-to-metal bonding was used, and various processing methods were investigated based on the type of packaging glass and the metal materials. Two typical bonding techniques are compression bonding and reactive bonding. The compression bonding utilizes different coefficients of thermal expansion of the materials to make the materials tightly squeezed together, while reactive bonding uses chemicals as bonding media. In the later development, ceramic-to-metal bonding techniques were developed and can be categorized into feedthrough (Forde and Ridgely, 2006), active brazing (Agathopoulos et al., 1997), non-active brazing (Messler, 2004), and diffusion bonding (Savage, 2013). With these bonding technologies, the electrode can pass through the ceramic encapsulation layer without affecting hermeticity. For example, Borton's group (Borton et al., 2013) integrated a 104 channel recording with a wireless neural interface using hermetic feedthrough assembly, which contains an array of 104 Pt Lr feedthrough pins embedded in groups of 8 metal-ceramic seals.

With the continuous advancement of hermetic bonding and sealing methods, various packaging materials are also emerging, such as SiO_2_ (Cheng et al., 2013b; Lee et al., 2013b; Song et al., 2019b; Chiang et al., 2020), Si_3_N_4_ (Oh et al., 2003; Zhao et al., 2019), SiC (Lei et al., 2016; Saddow et al., 2016), alumina (Al_2_O_3_) (Stieglitz, 2010; Shen and Maharbiz, 2019), aluminum nitride (AlN) (Murphy, 2008; Besleaga et al., 2017), and so on. Among these materials, SiO_2_ and Si_3_N_4_ have good chemical stability and unique optical properties. Particularly, SiO_2_, with internal transmittance is higher than 90% between 470 and 800 nm (Wang et al., 2011), has been utilized in the packaging of implantable devices that requires a certain degree of light transmission (Kino et al., 2018). For example, Song et al. reported a scalable approach for flexible biocompatible electronic systems, where thin microscale device components are integrated on a flexible polymer substrate to form an interconnected array for multimodal, high-performance biointerfaces (Song et al., 2019a). A thin SiO_2_ layer of 900 nm thermally grown on the surfaces of the silicon wafer served as an encapsulation layer. The SiO_2_ packaging at this thickness can provide a long-lived, flexible biofluid barrier for flexible devices. As an alternative, Al_2_O_3_ is not only chemically inert but also transparent at ultrasonic frequencies (Shen and Maharbiz, 2019), capable of packaging acoustic-based wireless medical devices wherein ultrasonic waves are used for efficient energy transfer and communication (Denisov and Yeatman, 2010; Seo et al., 2013). In recent years, SiC has become a hot topic in the packaging field because of its good biocompatibility and chemical inertia. SiC can be deposited at temperatures of lower than 400°C through plasma-enhanced CVD (PECVD) or low-pressure chemical vapor deposition (LPCVD) (Cogan et al., 2003; Hsu et al., 2007; Phan et al., 2019), making it compatible with the fabrication processes of many devices and materials. SiC package also provides less degradation rate in saline and better stability compared to Si_3_N_4_ and low-temperature SiO_2_ package (Lei et al., 2016). As evident in Kim et al.'s study (Kim et al., 2009), a multi-level hybrid packaging method based on PECVD deposited a-SiC_x_:H exhibited superior biocompatibility and reliability after accelerated lifetime testing. Furthermore, thin SiC films can become very flexible, suitable for use in packaging flexible implantable devices, such as ECoG arrays (Diaz-Botia et al., 2017).

Despite many advantages, the drawbacks of ceramic and glass materials cannot be ignored. First, even though most ceramics have good chemical stability, degradation of ceramics will still occur when the materials are soaked in ionic liquid environments, for example, Al_2_O_3_ dissolution in water. Second, there is a lack of viable etching techniques for ceramic and glass. Although many methods have been developed, the construction of ceramic and glass structures is still relatively complicated, making the package miniaturization difficult and incompatible with device fabrication technologies (Scholten and Meng, 2015). Third, the fabrication process of ceramic and glass package must be controlled precisely since even a small deposition variation can result in significant changes in package stability (Shen and Maharbiz, 2020).

### Soft Packaging Materials

Herein soft packaging materials generally refer to materials whose Young's moduli are between 10^5^ Pa (for soft tissue) and 10^10^ Pa (for hard tissue). It must be pointed out that the dividing line between flexible and solid packaging materials is changeable, solid packaging materials can also become flexible under certain conditions, such as small sizes, thin-film configurations, special structures, etc. (Viana et al., 2010). Compared to solid materials, soft polymeric materials dominate the choice of packaging materials for miniaturized neural implants because they offer many advantages, including high conformability, mechanical flexibility, small form factor, low price, and ease of use. Polymers can be cast, photopatterned, or dry etched at low temperatures, reducing the complexity of etching steps and infrastructure needs (Kim and Meng, 2015). Polymers also play an important role in the mechanical shielding of wire connectors to prevent accidental circuit breaks and provide a certain degree of mechanical buffering that avoids damaging the soft tissue by internal hard materials (Wasikiewicz et al., 2013). Many polymers have been developed and used to package neural implants, such as PI, PA, PDMS, polymethylmethacrylate (PMMA), liquid crystal polymers (LCPs), polycarbonate (PC), polystyrene (PS), SU-8, and so on. Due to their relatively high gas permeability (low hermeticity), thick polymer encapsulation must be used in chronic implants to protect the internal devices from being damaged (Jiang and Zhou, 2009; Wasikiewicz et al., 2013), at the expense of increased volume of the device and unstable thermal properties of the polymer (Barrese et al., 2013; Takmakov et al., 2015; Caldwell et al., 2020).

Among the emerging polymer packaging materials, PDMS is the most widely used coating material (Wasikiewicz et al., 2013) and the most established polymer for neural implants (Yoda, 1998; Colas and Curtis, 2004; Mata et al., 2005; Lacour et al., 2010; Rogers et al., 2010; Alt et al., 2016). PDMS offers good insulation, vibration absorption, good adaptability to tissue's deformation due to excellent elasticity (Wu et al., 1999; Kim et al., 2011; Minev et al., 2015; Alt et al., 2016), diffusional resistance to contamination solutes (Wu et al., 1999), good optical transparency (Jeong et al., 2015), hardly observed degradation (Alt et al., 2016), lower foreign body response (Bae et al., 2014), as well as low cost and availability. The most notable quality of PDMS is its superior, FDA-approved biocompatibility (Henle et al., 2011a; Bae et al., 2014) for chronic implants (USP class VI). It is one of the few packaging materials that have been tested for long-term implantation (Brindley et al., 1986; Schiavone et al., 2018). However, the high permeability of PDMS coating remains unsolved. A thin coating of PDMS cannot provide effective protection and may cause a delamination problem (Kinloch, 2012). Although a thick PDMS coating of 100–300 μm has significantly reduced permeability (Ordonez et al., 2012), the bulky material greatly restricts the miniaturization of the device, and thus, the utilization of PDMS in ultra-small implants. To address this challenge, attempts have been made by combining PDMS with other flexible materials such as PA (Henle et al., 2011a), PET (Shur et al., 2020) or PI (Ordonez et al., 2013) to form a composite packaging layer with improved hermeticity.

As an alternative polymer packaging material for long-term implants, Parylene consists of various chemical variants, including PA, Parylene D, Parylene HT, Parylene N, among which PA is one of the most prevailing packaging materials for neural implants (Ceyssens and Puers, 2015). It is also worth noting that Parylene HT is becoming more and more popular due to its improved packaging performance (Kumar, 2010). Currently, the commercial market of Parylene is dominated by two companies, Specialty Coating System (SCS) and Kisco Conformal Coating LLC (Kim and Meng, 2015). PA can be conformally deposited by CVD at room temperature and structured by oxygen plasma dry etching or laser. Those low fabrication requirements make PA compatible with many materials (Fan et al., 2020) and device designs. As a packaging material, PA has excellent biocompatibility (USP class VI), chemical inertness (De la Oliva et al., 2018), low conductivity, low intrinsic stress (Zöpfl et al., 2009), low pin-hole density, and conformal coating (Rodger et al., 2008). PA is also optically transparent with the transmission of 65–80 % over a spectrum range of 470 to 850 nm (Kwon et al., 2013; Alt et al., 2016; Bi et al., 2016), applicable for packaging many optical devices (Ledochowitsch et al., 2011; Park et al., 2014). However, PA has a low glass transition temperature (Tg = 90°C) (Kahouli et al., 2012), which limits subsequent fabrication methods. Although PA can effectively isolate external erosion for a certain period, long-term *in vivo* and reactive accelerated aging (RAA) studies show that the insulation properties of PA can degrade over time due to moisture absorption in liquid environments (Ordonez et al., 2012; Gong et al., 2020). A more in-depth understanding of PA's degradation mechanism will be critical for further improvement in the packaging performance of PA (Caldwell et al., 2020).

As a material with a long and rich history, the first discovery of PI can be traced back to 1908. Today, PI is already a very mature material and widely available in various forms (Liang et al., 1992; Rousche et al., 2001; Hassler et al., 2011; Bakonyi et al., 2013; Kim et al., 2013). PI has a great potential for a variety of applications in neural implants (Mian et al., 2005; Rubehn and Stieglitz, 2010; Viventi et al., 2011; Schaubroeck et al., 2017; Kampasi et al., 2020), such as multi-level interconnects, multi-chip module packaging, and flexible circuitry (Frazier, 1995). Compared with PA, PI provides better high-temperature stability (up to 400°C), higher glass transition temperature (Kim and Meng, 2015), better dielectric properties (Frazier, 1995), and lower moisture absorption. Especially for mechanical properties, PI has a tensile strength of 390 MPa, almost 6 times higher than that of PA, and Young's modulus of 8.37 GPa, 2.6 times higher (Stieglitz et al., 2000; Hassler et al., 2011). Consequently, PI enables much better durability under repetitive bending at the same thickness. Another advantage of PI is that its thermal expansion coefficient matches with Si so that thermally-induced mechanical stress can be negligible (Ceyssens and Puers, 2015). However, although PI has been proven to have considerably good biocompatibility, it is not FDA certified for human implantation. The poor adhesion of PI with certain materials, such as copper, is another major challenge (Kim and Meng, 2015; Bang, 2016). Moreover, studies show that the insulation lifetime of PI is quite limited in the saline environment, which may limit the use of PI in long-term implants. It is of note that significant shrinkage (20–50%) occurs during the PI curing process (Bagolini et al., 2002; Ma et al., 2009), which should always be considered in device design and manufacturing.

There are other polymer candidates in addition to the above materials. For example, PMMA exhibits higher impact resistance and lower electronic fluctuation (Joung, 2013; Kim et al., 2015) than PDMS and is expected as a possible replacement for PDMS in the future. SU-8 is a negative photoresist, epoxy-based polymer, which allows convenient, rapid, and cost-effective microfabrication processing (Márton et al., 2020). Despite the debate of the biocompatibility of SU-8 (Márton et al., 2020), SU-8 is still used as a packaging layer for many neural implants (Hong et al., 2018; Lee et al., 2019). For example, Hong et al. reported a SU-8 encapsulated, syringe-injectable mesh electronics, which enables multiplexed and chronically stable recording from diverse retinal ganglion cell (RGC) types in mice. LCPs have been explored as a stable and biocompatible material for both flexible substrates and packaging materials under *in vivo* conditions (Hwang et al., 2013). Besides their good chemical stability and high interfacial adhesion (Hwang et al., 2013), LCPs have lower permeability (2.19 × 10^−11^ atm cm^2^/s) than other polymer packaging materials (Au et al., 2019). Due to the surface alignment of the LCP when exposed to shear flow during fabrication, LCP has an anisotropic molecular structure and a crystalline surface with an amorphous core, resulting in relatively better mechanical and moisture barrier properties (Au et al., 2019). Although LCP has many attractive advantages, the long-term reliability of the LCP package still needs to gain widespread acceptance (Jeong et al., 2016). Moreover, due to its anisotropic structure, a small mistake in fabrication may cause tear-out, internal cracking, and other problems on the LCP surface, which makes further processing very challenging (Au et al., 2019).

### Biodegradable Encapsulation and Stiffener Material

A biopolymer usually is produced by microbial systems, extracted from plants, or chemically synthesized by biological components (Rebelo et al., 2017). Compared with synthetic polymers, the biggest advantage of biopolymers lies in their degradability and renewability (Cziple and Marques, 2008; Niaounakis, 2015). Generally speaking, the encapsulation and substrate material of chronic neural implants should remain stable in the host body. This requirement seems to contradict the degradability of biopolymers. However, it must be noted that some applications only require stable biotic-abiotic interfaces for a certain period of time (Muskovich and Bettinger, 2012; Lu et al., 2020). In the area of biodegradable encapsulation, Choi et al. (2020) demonstrated a bioresorbable polyanhydride-based polymer (PBTPA). The result shows PBTPA film has good biocompatibility, low swelling during dissolution in water, mechanical properties supporting robust operation in flexible devices, and good processability potential. By controlling the monomer composition and thickness, PBTPA can provide a strong water-barrier at timescales from hours to weeks. Moreover, the use of partial bioresorbable LED circuits illustrated its potential application in the optogenetics area. In addition to biodegradable polymers, Si membranes have also been explored as a water barrier layer for biodegradable encapsulation of neural interfaces. For example, John A. Rogers' group (Lee et al., 2017b) presented the use of silicon nanomembranes as bioresorbable water barriers in temporary electronic implants and environmental monitors.

Other major applications of the biopolymers include surface functionalization of the package surface to effectively reduce the immune response or temporary stiffening sheaths to escort a flexible implant into a designated position (Chung et al., 2019). At present, poly lactic acid (PLA), silk, and PEG are commonly used. Made from natural starch, such as corn, rice, and potatoes (Rebelo et al., 2017), PLA has relatively good mechanical properties and absorbability, and therefore are typically used in orthopedic devices, such as stents or scaffolds. The application of PLA does not stop at orthopedic study, as it can also be configured as a foam with oriented inner channels for repairing chronic spinal cord injuries (Cai et al., 2007). As an ancient material, the core silk fibroin fibers in raw silk have strong mechanical resistance. Silk fibroin and its other variants can be used in various soft tissues, such as ligaments (Altman et al., 2003), bladder (Franck et al., 2013), and musculoskeletal (Meinel and Kaplan, 2012). Recently, silk fibroin has been explored as the substrate material for optical or wirelessly powered neural implants (Hwang et al., 2012; Tao et al., 2015; Perotto et al., 2017). In the study of a flexible fish-bone-shaped neural probe (Wu et al., 2011), a silk sheath was utilized to reinforce the PI probe and provide temporary mechanical strength during probe implantation. Alternatively, PEG-based hydrogels hold tremendous promise as coatings to enhance the biocompatibility of neural prosthesis (Rao et al., 2011). For example, Kato et al. (2006) reported a multichannel flexible neural probe, in which the bioactive components of nerve growth factor (NGF) and PEG were mixed in the packaging material to repair the damaged neurons. PEG, another biodegradable polymer, has been utilized as a temporary stiffening sheath covering flexible electrode implants (Felix et al., 2013) or a stiffening filler in microfluidic channels of a flexible probe to improve the stiffness without increasing volumetric structure (Takeuchi et al., 2005). More comprehensive reviews of coating and stiffening materials are given in Kuo et al. (2013) and Wang et al. (2020).

## Conclusion and Outlook

Investigation of the novel electrode and packaging/substrate materials is, at present, one of the most prevailing topics in developing advanced neural recording electrodes, as evident by continuous growth in literature. While electrodes are the most significant element that directly influences the quality of neurophysiology recording, electrode packaging is equally important that help assist in device implantation as well as maintain device functionality and long-term stability. With recent advancements in material science and engineering, implantable electrode interfaces capable of recording neural activity with high spatiotemporal resolution can now be achieved. This article reviewed typical electrode and packaging materials associated with the state-of-the-art electrode devices, as guidance for future device development. Tables 1, 2 summarize the main properties of the selected electrode materials and packaging materials, respectively. In particular, Table 1 lists the properties of various electrode materials discussed in this review, including their electrical properties, biocompatibility, stability, biodegradability and bioresorbability, mechanical flexibility and bendability, Young's modulus and broad-band optical transmission, as detailed in sections Key Material Characteristics and Electrode Materials. Table 2 discusses the water vapor permeability, Young's modulus, optical transparency from 470 to 800 nm, and stability of the representative packaging materials introduced in this article. Of these materials, synthetic polymers have the most balanced performance and can maintain good packaging performance for a relatively long time.

**Table 1:** Summary of various electrode materials with key properties.

**Electrode materials**		**Electrical properties (impedance @1 kHz)**	**Biocompatibility**	**Stability**	**Biodegradability/ Bioresorbability**	**Mechanical flexibility/ be-ndability**	**Young's modulus**	**Optical Transmission (400–700 nm)**	**References**
	Nanopillars	13.1 ± 2.7 k*Ω*-1172.3 ± 241.6 k*Ω* (0–22.5 μm height)		14 days *in vitro*					
GNPs	Nanorods	1.847 k*Ω* (10,000 μm^2^ area)	Cytotoxic (depend on the size of GNPs)	20 times (agarose gel insertion)	Biodegradable	Bendable	1–10 GPa	Opaque	Zhou et al., 2009; Kim et al., 2010b; Nick et al., 2014; Lee et al., 2016b
	Nanoflakes	11.9 ± 1.47 k*Ω*-249 ± 28.1 k*Ω* (5–50 μm diameter)		A month					
Pt black		3.5 k*Ω* (4 mm length, ~100 μm diameter)	Biocompatible	3 days *in vivo*	N/A	Bendable	N/A	Opaque	Zhang et al., 2015; Lee et al., 2017a; Zátonyi et al., 2018
Au/Pt alloy		0.23 M*Ω* (20 nm diameter)	Biocompatible	7 times (ultrasonic treatments)	N/A	N/A	113.8 GPa	Opaque	Zhao et al., 2016
Ir/Pt alloy		80 ± 18 k*Ω* (13 mm length, 75 μm diameter)	Biocompatible	12 weeks *in vivo*	N/A	Bendable	185.5–189.6 GPa	Opaque	Cassar et al., 2019
Si nanowires		~20 M*Ω* (100 nm−200 nm tip diameter)	Biocompatible	8 days (rodent neurons) 6 weeks (hiPSC-derived neurons)	Biodegradable	N/A	60–240 GPa	Transparent	Sohn et al., 2010; Marcon and Boukherroub, 2014; Liu et al., 2017a
Si NMs		~50 k*Ω*-~250 k*Ω* (200^2^-500^2^ μm^2^)	Biocompatible	A month *in vivo*	Bioresorbable	Flexible	3.25–180 GPa (2 nm−25 nm thickness)	Transparent	Yu et al., 2016; Bai et al., 2019
ITO/PEDOT:PSS		~ 40 k*Ω*-~100 k*Ω* (10–80 μm diameter)	Biocompatible	4 weeks *in vitro*	N/A	Flexible	~77 GPa (on glass)	Transparent (> 80 %)	Li and Chang, 2014; Yang et al., 2017
PEDOT:PSS/nanostructur-ed Pt		9.2 k*Ω* (500 μm diameter)	Biocompatible	1,500 CV cycles	N/A	Flexible	N/A	Opaque	Boehler et al., 2017
PPy nanotubes/GNP		~5 k*Ω* (300 μm diameter)	Biocompatible	Stable	Biodegradable	Flexible/benda-ble	N/A	N/A	Kojabad et al., 2019
Diamond		~ 207.9 k*Ω* (0.0079 mm^2^ area)	Biocompatible	Stable	N/A	Flexible (on Parylene C)	~10^3^ GPa	Opaque	Fan et al., 2020
Graphene		243.5 ± 15.9 k*Ω* (~200 μm diameter)	Biocompatible	70 days *in vivo*	N/A	Flexible	~1 TPa	Transparent (>90%)	Lee et al., 2013a; Park et al., 2014
CNFs		~1 M*Ω* (2 cm length, 25.7 ×16.6 μm^2^)	Biocompatible	4 weeks *in vivo*	Unbiodegradable	Flexible	6–207 GPa	N/A	Lawrence et al., 2008; Guo et al., 2017; Farzamfar et al., 2019
CNTs		~64.5 *Ω* mm^−2^	Biocompatible	Stable	Unbiodegradable	Flexible	530–700 GPa	Transparent (~60%)	Lawrence et al., 2008; Su et al., 2010; Deng et al., 2011
Glassy carbon		11.0 ± 5.4 k*Ω* (300 μm diameter)	Biocompatible (12 days)	Stable	N/A	Flexible	20 GPa	Opaque	Vomero et al., 2016, 2017

**Table 2:** Summary of various packaging materials with key properties.

	**Permeability (H_**2**_O)($\frac{{\text{cm}}_{\text{STP}}^{3}•\text{cm}}{{\text{cm}}^{2}•\text{s}•\text{cmHg}}$) at 20–35^**°**^C**	**Young's modulus (GPa)**	**Transparency** **From wavelengths 470 nm-800 nm (@wavelength) (thickness)**	**Stability** ***in vivo*** **or in solution**		**References**
				**Time**	**Method**	
Titanium (thin film)	≈0	90	~55% (5 nm)	16 years	*In vivo*	Scarano et al., 2005; Greenhouse et al., 2011; Nakai et al., 2011; Axelevitch et al., 2012
platinum	≈0	213	~35% (20 nm)	3.25 years	*In vivo*	Farraro and Mclellan, 1977; Oh et al., 1993; Griffith and Humphrey, 2006; Greenhouse et al., 2011
SiO_2_	4.63 E-16	66	91–88% (1 mm)	~60 year (Converted to 37°C)	PBS Soak (95°C)	Jaccodine and Schlegel, 1966; Fahlteich et al., 2011; Wang et al., 2011; Song et al., 2019a
Si_3_N_4_	2.06 E-25	319.4	15% (@450 nm)-60%(@800 nm) (1 mm)	383 days	*In vivo*	Bruls et al., 2001; Su et al., 2004; Wise et al., 2004; Andringa et al., 2015
SiC	6.18 E-21	410	90% (@450 nm) (300 nm)	> 6 weeks	*In vivo*	Anma et al., 2001; Chawla et al., 2004; Zambov et al., 2006; Vomero et al., 2018
Al_2_O_3_	1.73 E-16	303	85% (@450 nm)-0% (700 nm) (1 mm)	>5 months	PBS Soak (37°C)	Vekinis et al., 1990; Jiang et al., 2008; Fahlteich et al., 2011; Peled et al., 2014
PI	6.35 E-7	8.45	80% (25 μm)	1,091 days	*In vivo*	Hubbell Jr. et al., 1975; Rubehn and Stieglitz, 2010; Barrese et al., 2013
PA	1.9 E-7	4.75	95% (20 μm)	1,200 days	*In vivo*	Hubbell Jr. et al., 1975; Shih et al., 2003; He et al., 2009; Barrese et al., 2013
PDMS	4 E-5	7.5E-4	93.39%	>18 weeks	*In vivo*	Armani et al., 1999; Metz et al., 2005; Henle et al., 2011b; Ko et al., 2017
PMMA	11.4 E-9	2	94%	3-6 months	*In vivo*	Kim et al., 2004; Jackson et al., 2010; Landi et al., 2013; Keller and Kouzes, 2017
LCPs	1.14 E-11	10	50% (@650 nm)- 90% (@850 nm)	2.5 years	*In vivo*	Mehta and Isayev, 1991; Flodberg et al., 2000; Jeong et al., 2016
CS membrane	2.4 E-3	0.013	70% (@450 nm)-83%(800 nm) (0.5 um)	120 days	*In vivo*	Kweon et al., 2001; Gu et al., 2013; Li et al., 2014; Meyer et al., 2016
Silk fibroin film	1.2 E-3	0.034	90%	2 weeks	*In vivo*	Kweon et al., 2001; Hopkins et al., 2013; Cho et al., 2014
PLA	2.4 E-10	4.2–5.7	92–94%	4 months	*In vivo*	Solarski et al., 2005; Bang and Kim, 2012; Tyler et al., 2016; Arrieta et al., 2017

With the trend of further miniaturization in large-scale, high-density recording electrodes, many challenges still remain unsolved, mostly related to chronic stability, high fidelity of recording, and minimal foreign-body immune responses. For moving forward, one research area that has received much recent attention is to design and develop composite materials that combine the unique advantages of different existing materials while eliminating their major drawbacks. The use of composite materials in electrode structuring has the potential to bring disruptive changes to single material designs. For example, Yang et al. designed a PEDOT:PSS-ITO-Ag-ITO on PA assembly that greatly enhanced the transparency and electrochemical conductivity while overcoming the brittleness of ITO and the oxidation of Ag thin films. Pal et al. (2016) demonstrate a flexible bio-sensor that combines PEDOT:PSS sensing elements on a fully biodegradable and flexible silk protein fibroin support to achieve excellent electrochemical activity and stability over days. Composite electrode materials can be prepared by *in situ* electrodeposition or multilayered assembly of inorganic and/or organic conducting materials on planar substrates to achieve the desired electrochemical, biological, optical, and mechanical properties. Recently, with their tunable composites, configurations, and density, 3D nanostructured materials represent novel electrode materials to further improve the electrochemical impedance and the capacity of the injection charge density, two important factors that determine the SNRs and recording quality of the electrodes. While promising, the *in vivo* evaluation of these composite materials is incomplete, preventing their applications in chronic neural interfaces.

Surface modification combining the traditional materials (such as PA, PI, Ceramic) with biopolymers or nanomaterials also greatly expands the potential application scenarios of packaging materials. As an important technology, surface functionalization can be achieved by fabricating nanofibers from synthetic polymers and biopolymers with different bioactive molecules to improve their applicability (Sofi et al., 2019). Common techniques include but not limited to electrospinning (Barakat et al., 2010), plasma treatment (Grace and Gerenser, 2003), wet chemical treatment (Nam et al., 1999), surface grafting (Liu et al., 2004), etc. These surface functionalization techniques can modify the packaging material with varying mechanical stiffnesses and improved biocompatibility according to specific needs (Ghasemi-Mobarakeh et al., 2009; Sofi et al., 2019). However, the long-term stability, scalability, and compatibility of these surface functionalization techniques with other electrode fabrication/packaging techniques remain unclear and deserve further investigation.

Besides improving existing materials, new electrode materials (e.g., diamond and MXenes) and structure/packaging materials (e.g., self-healing polymer and shape memory polymer) that were not originally used in neural interfaces are being explored (Driscoll et al., 2018, 2020). For example, Driscoll et al. purposed flexible Ti_3_C_2_ MXene microelectrode arrays for *in vivo* micro-ECoG recording with the benefits of significantly high volumetric capacitance, electrical conductivity, surface functionality, and sensitivity (Driscoll et al., 2020). As an emerging packaging material, the self-healing materials, such as a self-healing PDMS-based elastomer, have been explored to build self-healing, flexible electrodes (Dhler et al., 2020), which has a potential application in neural implants. Most recently, Bashandeh et al. reported an SMP material as a precursor to form different 3D kirigami microstructures (Bashandeh et al., 2020). While significant progress has been made, comprehensive evaluation of their functionality, long-term stability and biocompatibility is needed to fully realize the true potential of these new materials for use in neural recording interfaces.

## Author Contributions

WY and YG wrote the manuscript. WL revised the manuscript. All authors contributed to the article and approved the submitted version.

## Conflict of Interest

The authors declare that the research was conducted in the absence of any commercial or financial relationships that could be construed as a potential conflict of interest.

## Glossary

EEG, Electroencephalogram; ECoG, Electrocorticogram; LFPs, Local field potentials; SNR, Signal-to-noise ratio; ITO, Indium tin oxide; SiC, Silicon carbide; PDMS, Polydimethylsiloxane; LCPs, Liquid crystal polymers; PEDOT:PSS, Poly(3,4-ethylene dioxythiophene):poly(styrene sulfonate); PPy, Poly(pyrrole); ROS, Reactive oxidative species; CPs, Conducting polymers; BDD, Boron-doped polycrystalline diamond; Mo, Molybdenum; ISO, International Organization of Standard; PEG, Polyethylene glycol; PEGMA, Polyethylene glycol methacrylate; GNPs, Gold nanoparticles; Si NM, Silicon nanomembranes; SiO_2_, Silicon dioxide; PBS, Phosphate-buffered saline; DI, De-ionized; PA, Parylene C; PI, Polyimide; SMPs, Shape-memory polymers; Au, Gold; PET, Polyethylene terephthalate; Pt, Platinum; Ir, Iridium; W, Tungsten; Ta, Tantalum; 3D, Three dimensional; 2D, Two dimensional; IrOx, Iridium oxide; EPIC, Electrodeposited a PtIr coating; Si, Silicon; CMOS, Complementary metal-oxide-semiconductor; FETs, Field-effect transistors; PLGA, Poly(lactic-co-glycolic acid; Ge, Germanium; SiGe, Silicon germanium; IZO, Indium-doped zinc oxide; a-IGZO, Indium-gallium-zinc oxide; ZnO, Zinc oxide; Ra, Average surface roughness; PANi, Poly(aniline); PT, Poly(thiophene); CVD, Chemical vapor deposition; CFs, Carbon fibers; CNTs, Carbon nanotubes; CFMEs, Carbon fiber microelectrodes; CNF, Carbon nanofiber; COC, Cyclic olefin copolymer; fMRI, Functional magnetic resonance imaging; SWCNTs, Single-walled carbon nanotubes; MWCNTs, Multi-walled carbon nanotubes; GC, Glassy carbon; EM, Electromagnetic; Al_2_O_3_, Alumina; AlN, Aluminum nitride; PECVD, Plasma-enhanced chemical vapor deposition; LPCVD, Low-pressure chemical vapor deposition; PMMA, Polymethylmethacrylate; PC, Polycarbonate; PS, Polystyrene; RAA, Reactive accelerated aging; SCS, Specialty Coating System; RGC, Retinal ganglion cell; PBTPA, Bioresorbable polyanhydride-based polymer; PLA, Poly lactic acid; NGF, Nerve growth factor.
